# Tumor Microenvironment Landscapes Supporting EGFR-mutant NSCLC Are Modulated at the Single-cell Interaction Level by Unesbulin Treatment

**DOI:** 10.1158/2767-9764.CRC-23-0161

**Published:** 2024-03-26

**Authors:** Giorgia Maroni, Indira Krishnan, Roberta Alfieri, Valerie A. Maymi, Nicole Pandell, Eva Csizmadia, Junyan Zhang, Marla Weetall, Art Branstrom, Giulia Braccini, Eva Cabrera San Millán, Barbara Storti, Ranieri Bizzarri, Olivier Kocher, Daniela S. Bassères, Robert S. Welner, Maria Cristina Magli, Ivan Merelli, John G. Clohessy, Azhar Ali, Daniel G. Tenen, Elena Levantini

**Affiliations:** 1 Cancer Science Institute of Singapore, National University of Singapore, Singapore.; 2 Harvard Medical School, Boston, Massachusetts.; 3 Institute of Biomedical Technologies, National Research Council (CNR), Pisa, Italy.; 4 Beth Israel Deaconess Medical Center, Boston, Massachusetts.; 5 Preclinical Murine Pharmacogenetics Core, Beth Israel Deaconess Cancer Center, Dana-Farber/Harvard Cancer Center, Boston, Massachusetts.; 6 PTC Therapeutics, South Plainfield, New Jersey.; 7 NEST, Scuola Normale Superiore and Istituto Nanoscienze-CNR, Pisa, Italy.; 8 Department of Surgical, Medical and Molecular Pathology, and Critical Care Medicine, University of Pisa, Pisa, Italy.; 9 Biochemistry Department, Chemistry Institute, University of Sao Paulo, Sao Paulo, Brazil.; 10 Department of Medicine, Hemathology/Oncology, University of Alabama at Birmingham, Birmingham, Alabama.; 11 Harvard Stem Cell Institute, Cambridge, Massachusetts.

## Abstract

Lung cancer is the leading cause of cancer deaths. Lethal pulmonary adenocarcinomas (ADC) present with frequent mutations in the EGFR. Genetically engineered murine models of lung cancer expedited comprehension of the molecular mechanisms driving tumorigenesis and drug response. Here, we systematically analyzed the evolution of tumor heterogeneity in the context of dynamic interactions occurring with the intermingled tumor microenvironment (TME) by high-resolution transcriptomics. Our effort identified vulnerable tumor-specific epithelial cells, as well as their cross-talk with niche components (endothelial cells, fibroblasts, and tumor-infiltrating immune cells), whose symbiotic interface shapes tumor aggressiveness and is almost completely abolished by treatment with Unesbulin, a tubulin binding agent that reduces B cell–specific Moloney murine leukemia virus integration site 1 (BMI-1) activity. Simultaneous magnetic resonance imaging (MRI) analysis demonstrated decreased tumor growth, setting the stage for future investigations into the potential of novel therapeutic strategies for EGFR-mutant ADCs.

**Significance:**

Targeting the TME is an attractive strategy for treatment of solid tumors. Here we revealed how EGFR-mutant landscapes are affected at the single-cell resolution level during Unesbulin treatment. This novel drug, by targeting cancer cells and their interactions with crucial TME components, could be envisioned for future therapeutic advancements.

## Introduction

Lung cancer, the deadliest malignancy worldwide, accounts for 30% of tumor-related deaths (1). Irrespective of early remission and overall improvement of patient outcomes, resistance to currently available therapeutics inevitably develops, thus necessitating improved treatment options for lung cancer.

Non–small cell lung cancer (NSCLC) accounts for approximately 85% of lung cancer cases (2), and mutant EGFR is one of its most common oncogenic drivers (10%–40%), which is associated with poor prognosis and limited therapeutic efficacy (3–5). Currently, patients with advanced NSCLC harboring “classic” EGFR mutations (Ex19Dels and L858R) usually receive tyrosine kinase inhibitors (TKI) as the standard first-line treatment (5). Clinical trials directly comparing first-generation (gefitinib, erlotinib), second-generation (afatinib), and third-generation (osimertinib) EGFR TKIs, demonstrated that osimertinib extended both progression-free survival and overall survival (6). The “uncommon” EGFR mutations occurring in exon 18–21 may respond to gefitinib, erlotinib, or afatinib, while exon 20 insertions or *de novo* T790M mutations are resistant to first- and second-generation EGFR TKIs (5, 7–12). Furthermore, for patients who received first- or second-generation EGFR TKIs as first-line treatment and developed the EGFR T790M resistance mutation at disease progression, osimertinib can be administered as second-line option (5, 13, 14).

To gain insights into the crucial events of lung cancer development, we adopted as prototype of aberrant EGFR activation a key genetically engineered murine model (GEMM) of NSCLC, carrying two recurrent activating mutations identified in patients (15), that is, the *L858R-T790M* transgenic mice (EGFR^TL^ mice; 16).

Tumors are composed of different cell types whose transcriptional programs, and interactions with other cells, constituting the tumor microenvironment (TME), are subject to intricate control mechanisms and play critical roles in tumor progression and treatment response. Despite the significant disruption caused by EGFR mutations in lung cancer, their oncogenic consequences on transformed epithelial subpopulations still require investigation. In addition, the intercommunications occurring within distinct TME components and nearby individual cancer subpopulations, are so far undescribed. Moreover, how these interplays evolve and interact longitudinally in response to therapeutic treatment is unknown, as much of this complexity is lost in traditional bulk RNA-sequencing investigations.

Conversely, single-cell RNA sequencing (scRNA-seq) can provide high-definition evaluation of intratumor heterogeneity, by capturing multidimensional information unbiasedly, and clarifying cellular identity by grouping cells according to their gene expression profiles. Thus, by means of droplet-based molecular barcoding techniques, we analyzed single-cell transcripts and deconvoluted the distinct cell type populations within the heterogeneous tumor cellular background of EGFR^TL^ mice, thereby identifying which cellular subpopulations exist during malignant growth and how they respond to therapy. Finally, by utilizing computational inference modeling, we studied receptor-ligand communication between cells to assess their cross-talk within the TME during tumor evolution and treatment. Our data identified (i) a unique cellular atlas of healthy lungs and EGFR^TL^-driven adenocarcinomas (ADC), by resolving distinct epithelial and TME clusters/subpopulations of immune, endothelial, and fibroblast nature; (ii) new cell subtypes that are distinguishingly associated with EGFR-driven lung tumors, highlighting differences between tumor and healthy tissue; (iii) a comprehensive single cell–based ecosystem of cell-cell communications contributing to mold the aggressive ADC environment.

EGFR-mutant NSCLCs have become a paradigm to study tumor response to drug treatments, leading to significant improvements in clinical outcomes. The majority of EGFR-mutant patients, carrying either primary or acquired mutations (6), are treated with the third-generation TKI osimertinib; however, they all eventually relapse (17–22), and no standard therapy after osimertinib defeat has been established yet. Here, we tested in EGFR^TL^-driven ADCs the effects of Unesbulin (23, 24), a novel drug in phase Ib clinical trial (Identifier NCT02404480), identified for its ability to inhibit the activity of BMI-1, an oncogene involved in multiple types of tumors (24, 25). BMI-1 is a key component of the epigenetic complex PRC1 (polycomb repressive complex-1), belonging to the 11-gene death-from-cancer signature (26), and we previously demonstrated that is a critical gene in NSCLC (23, 25).

*In vivo* treatment of EGFR^TL^ mice carrying pulmonary ADCs demonstrated antitumor growth response to Unesbulin, as assessed by MRI.

Therefore, given the above, we adopted such model to perform a proof-of-concept study aimed at evaluating drug response at the single-cell level, not solely as dependent on the transformed epithelial cells, but also as largely molded through its interaction with the TME. We particularly focused on interactions between transformed epithelial cells and macrophages, the most abundant immune infiltrating population in addition to endothelial cells and fibroblasts. Our data highlighted that upon drug treatment pulmonary tumor growth is decreased, and a defined tumor-enriched epithelial subpopulation is affected by shutting down EGFR signaling and cell growth, while the normal epithelial components are virtually unaffected. In addition, we were able to demonstrate a major readjustment of TME components and their signaling during drug treatment, thus depicting the transcriptional dynamics encompassing tumor response to Unesbulin.

Our findings strongly encourage development of BMI-1–targeted therapies for patients with NSCLC carrying mutant EGFR, suggesting an exciting opportunity for precision medicine in the genetically complex NSCLC disease. In addition, by adopting predictive modeling, we inferred the intercommunications occurring between cancer cells and TME components. Tumor growth at homeostasis and during drug response served as a framework to delve into the dynamic cross-talk regulating the tumor milieu, whose relevance must be increasingly addressed to improve treatment response in complex clinical settings.

## Materials and Methods

### Cell Culture

The human lung cancer cell line H1975 harboring the *EGFR ^L858R/T790M^* mutation was purchased from ATCC. Cells were cultured in RPMI1640 medium containing 10% heat-inactivated FBS at 37°C in a humidified incubator with 5% CO_2_. These cells were authenticated via DNA fingerprinting (Supplementary Data S1) and tested negative for *Mycoplasma* (27).

H1975 cell line was used at passage 2 and it was treated with Unesbulin (1 µmol/L), PTC-028 (1 µmol/L), or Vehicle (0.5% DMSO) for various timepoints (24, 48, and 72 hours) and the lysates subjected to Western Blotting and FACS analysis.

### Murine Models

Mice were housed in a sterile barrier facility, and all experiments were approved by the Institutional Animal Care and Use Committee at the Beth Israel Deaconess Medical Center.

### Xenografts and Drug Treatment

To study the *in vivo* effect of Unesbulin or its analog PTC208 on H1975 cell lines, *NOD-SCID IL2Rγ*(null) mice (NSG mice, Jackson Laboratories) were injected subcutaneously in flanks on both sides with 1 × 10^6^ cells with 50 µL Matrigel (BD Basement Membrane Matrix Phenol red-free #356237). Once tumors became measurable, mice were randomized to receive Unesbulin (12 mg/kg; *n* = 17), PTC-028 (15 mg/kg; *n* = 8), or Vehicle (0.5% hydroxypropyl methyl cellulose—HPMC—and 0.2% Tween 80 in distilled water; *n* = 15) by oral gavage twice a week. To determine tumor volume by caliper measurement, the greatest longitudinal diameter (length) and the greatest transverse diameter (width) were determined. Tumor volume was calculated by the modified ellipsoidal formula [tumor volume = ½ (length × width^2^), as reported previously (23, 28–30). Treatment was started when tumor volume was at least 0.06 cm^3^, and tumor growth was followed up to 21 days.

### Transgenic Mice, Drug Treatments, and MRI

Generation of bitransgenic mice carrying the Clara cell secretory protein (*CCSP*)-*rtTA* and TetO-regulated *EGFR^L858R/T790M^* transgenes and genotyping were done as described previously (29, 31). The expression of mutant EGFR and development of lung tumors was induced by feeding 8 to 10 weeks old mice with doxycycline impregnated food pellets (0.625 g/kg Rodent diet TD.01306, Envigo) for 2 months. Control mice were either CCSP−/EGFR+ or CCSP+/EGFR− treated with doxycycline. Mice were then imaged by MRI at the BIDMC Imaging Facility every 2 weeks to detect baseline tumor volume and recruited into treatment groups (Unesbulin or Vehicle), when tumor size reached 1.5 to 2.0 mm^3^.

Mice were treated with Unesbulin (*n* = 8; 12 mg/kg in 0.5% HPMC and 0.2% Tween, oral gavage twice per week), or vehicle control (*n* = 6; 0.5% HPMC and 0.2% Tween, same regimen as Unesbulin).

Mice were then scanned by MRI twice per week to capture the effects of drug treatment on tumor size over a month period. Processing and quantification techniques of tumor burden were based on manual segmentation/volume calculation of diffuse lung tumors as described previously (23, 28–30, 32). Changes in lung tumor volumes over the course of treatment were calculated as percentage change in volume over tumor volume at day 1 of treatment, which was set at 100%. MRI images of mouse lungs were captured with a Bruker Biospec 94/20 9.4 Tesla scanner and the primary imaging sequence used was RARE (Rapid Acquisition with Refocused Echoes), with repetition time (TR)/echo time (TE) = 1,200 ms/17.5 ms.

### Histopathologic and Immunofluorescence Analyses

Mice were sacrificed by CO_2_ euthanasia. Lungs and xenograft subcutaneous tumors were fixed in 10% formalin (formalin solution neutral buffered 10%, Sigma-Aldrich) O/N. Fixed specimens were embedded in paraffin and sectioned at 5-µm thickness. Tissue sections were stained with hematoxylin and eosin, for pathology analysis. IHC were performed on paraffin sections with an anti-BMI-1 mouse monoclonal antibody (Millipore, #05637; 1/100 dilution) on mouse tissues. Briefly, tissue sections were deparaffinized with xylene and hydrated in graded ethanols. Antigen retrieval was performed in 10 mmol/L citrate buffer (pH 6.0) on a 2100 Retriever for 40 minutes. To prevent nonspecific binding, protein-blocking solution 7% horse serum in PBS was applied for 30 minutes at room temperature. Primary antibodies were incubated overnight at 4°C. Next, we applied peroxidase blocking solution for 10 minutes at room temperature and subsequently we performed secondary antibody incubation for 1 hour at room temperature. Secondary antibodies were horse anti-mouse BA2001 at 1/1,200 dilution, and goat anti-rabbit BA1000 at 1/1,000 from Vector Laboratories, Inc. ABC-HRP (horseradish peroxidase) standard kit (Vector Labs, CA PK-6100) was adopted and incubated for 30 minutes and the signal was revealed with 3,3ʹ-Diaminobenzidine (DAB) (Vector Labs, CA SK-4100). Tissue sections were counterstained with hematoxylin, dehydrated in graded ethanols, and xylene and mounted with Cytoseal 60 (Electron Microscopy Science).

### Immunofluorescence on Pulmonary Tissues

Paraffin-embedded tissues were sectioned at 5 µm thickness. Tissue sections were deparaffinized with xylene and hydrated in graded ethanols. Antigen retrieval was performed in pressure cooker for 10 minutes in 10 mmol/L citrate buffer pH 6.0. We applied protein blocking with 5% donkey serum in PBS for 30 minutes at room temperature. Primary antibody (Rabbit anti-human Ubiquityl-Histone H2A mAb; Cell Signaling Technology #8240S; 1:500 dilution) was incubated at 4°C overnight. After washing with PBS, sections were incubated with secondary antibody (Donkey anti-Rabbit IgG AlexaFluor 647 from Jackson ImmunoResearch #711-605-152 at 1:300 dilution) for 1 hour at room temperature. The slides were counterstained with Hoechst 33342 (Thermo Fisher Scientific #H3570) for nuclear staining after incubation with secondary antibody step.

After washing, tissue sections were mounted with ProLong Gold Antifade Mountant (Thermo Fisher Scientific #P36930) and fluorescence was measured using confocal Zeiss LSM 880 with Airyscan (Carl Zeiss) and Nikon TiE2 supplied with ViCo sectioning system (Nikon).

### Western Blot Analysis

H1975 cells were lysed directly in-plate with RIPA buffer [20 mmol/L Tris-HCl (pH 7.5), 150 mmol/L NaCl, 1 mol/L Na_2_ ethylene diamine tetracetic acid (EDTA), 1 mmol/L ethylene glycol diamine tetracetic acid (EGTA), 1% NP-40, 1% sodium deoxycholate, 2.5 mmol/L sodium pyrophosphate, 1 mmol/L β-glycerophosphate, 1 mmol/L Na_3_VO_4,_ 1 mmol/L phenylmethylsulfonylfluoride and cOmplete EDTA-free Protein Cocktail for protease inhibitors and PhosStop as phosphatase inhibitor cocktail. The lysates were centrifuged at 13,000 × *g* for 15 minutes at 4°C, the supernatant was snap frozen in liquid N_2_ and stored at −80°C. For xenografts and mouse lung tumors, tumors were snap frozen in liquid N_2_, lysed (in RIPA buffer) and homogenized in Dounce homogenizer on ice. The supernatant was saved after spinning at 13000 × *g* for 15 minutes at 4°C. A total of 15 mg of total protein were separated on 4%–20% SDS-PAGE gels and transferred to polyvinylidene difluoride [or Nitrocellulose) membrane using the TransBlot Transfer System (Bio-Rad). Membranes were blocked in 5% nonfat milk/Tris-buffered Saline with Tween 20 (TBST)] and incubated with primary antibodies against Bmi-1 (1:1,000 Cell Signaling Technology #6964S) or ubiquityl-Histone H2A (Lys119; 1:2,000 Cell Signaling Technology #8240) antibodies overnight at 4°C. Membranes were then stripped with Restore Western Blotting Buffer Solution (Thermo Fisher Scientific #21059) for 15 minutes at room temperature and incubated overnight with an anti-β-actin mouse antibody (Santa Cruz Biotechnology #81178) at a 1:1,000 dilution to assess equal loading. The blots were incubated with specific HRP-conjugated secondary antibodies anti-rabbit (anti-rabbit-IgG-HRP Santa Cruz Biotechnology #SC2054) or anti-mouse (anti-mouse-IgG-HRP Santa Cruz Biotechnology #SC2031). In the case of mouse tissues, the secondary antibody used to detect β-actin was Mouse TrueBlot ULTRA: anti-mouse Ig-HRP (1:1,000; Rockland Immunochemicals Inc.18-8817-33). An enhanced chemiluminescence blotting analysis system (Pierce, Thermo Fisher Scientific #32106) was used for antigen-antibody detection. The density of Western blot bands was quantified by ImageJ software (Version 1.51m9, NIH, Bethesda, MD).

### Cell Cycle Analysis

A combination of Vybrant DyeCycle Violet and Pyronin Y was used for the differential staining of cellular DNA and RNA. H1975 cells treated with Unesbulin (1 µmol/L), PTC-028 (1 µmol/L) 1 or DMSO (0.5%) for 24 hours, were permeabilized in phosphate-citrate buffer solution (pH 4.8), washed, and then resuspended in a solution of 5 µmol/L Vybrant DyeCycle Violet (Thermo Fisher Scientific) and 4 µg/mL pyronin Y (Polysciences). Cycle status was then evaluated by flow cytometry on Cytoflex Flow Cytometer (Beckman Coulter, Inc.). FlowJo version 10.0 was used to analyze the cell cycle data.

### Lentivirus Production and Infection

Briefly, a lentivirus vector (CS-H1-shRNA-EF-1α-EGFP) expressing short hairpin RNA (shRNA) against human *Bmi-1* (target sequence CAGATGAAGATAAGAGAAT for sh-1; GAGAAGGAATGGTCCACTT for sh-2), and *Luciferase* was used (33), as described previously (25). HEK-293T cells, cultured in DMEM containing 10% heat-inactivated FBS and 1% Penicillin-Streptomycin, were cotransfected using TransIT Lenti Transfection Reagent (Mirus #MIR6600) with lentiviral packaging constructs (Gag-Pol and VSV-G Env). Virus was harvested and concentrated using Lenti-X Concentrator (Takara # 631232). A single lentiviral transduction was performed in culture dishes (Falcon 1008; Becton Dickinson) in the presence of Polybrene (8 µg/mL; Sigma #TR-1003-G).

### BMI-1 Knockdown Xenograft Assays

To study the *in vivo* effects of *Bmi1* knockdown on H1975 cells, NSG mice were transplanted with 270,000 cells transduced with two different GFP-coupled shRNAs against human *Bmi1* (sh1 *n* = 1, and sh2 *n* = 2), or with a control GFP-coupled shRNA (Luciferase-sh *n* = 1). Forty eight hours after, the H1975 cell line was infected with a multiplicity of infection = 20, GFP^+^ cells were FACS-sorted and then injected into the NSG mice. Tumor volumes were assessed by caliper (as described above) up to 17 days.

### Mouse Lung and Tumor Dissociation into Single Cells

Briefly, the mouse pulmonary tissue (healthy and tumor lung) was dissociated for scRNA-seq downstream applications using the Tumor Dissociation kit by Miltenyi Biotec (# 130-096-730). The tissue was placed in a petri dish on ice and cut into small pieces of 2–4 mm. The pieces were infused with RPMI/enzyme mix (Miltenyi Biotec), transferred to a gentleMACS C tube containing RPMI/enzyme mix, attached to the sleeve of the gentleMACS Octo Dissociator and run using a “37C_m_TDK_1” program. After termination of the program, the cells were spun down at 300 × *g* for 10 minutes at 4°C, resuspended in RPMI-2% FBS, passed through a 70 µm strainer ad centrifuge was repeated. The cell pellet was treated with 1 mL of ammonium chloride potassium (ACK) solution for 7 minutes at room temperature, the lysis stopped with 4 mL of RPMI-2% FBS. After centrifugation, the cells were suspended in 1 mL RPMI-2% FBS and passed through a 70 µm cell strainer to obtain a single-cell suspension, as reported previously (23, 34). Immediately before transcriptome barcoding using the inDrop platform, cells were manually counted on a hemocytometer and diluted to 60k cells/mL. The final cell suspension included 15% v/v Optiprep (Sigma-Aldrich, catalog no. D1556).

### InDrop

For Droplet-based single cell (scRNA) seq, the cells were encapsulated in droplets and the libraries were made following a previously described protocol (34, 35) at the Harvard Single Cell Core with the following modifications in the primer sequences.

RT primers on hydrogel beads-5′CGATTGATCAACGTAATACGACTCACTATAGGGTGTCGGGTGCAG[bc1,8nt]GTCTCGTGGGCTCGGAGATGTGTATAAGAGACAG[bc2,8nt]NNNNNNTTTTTTTTTTTTTTTTTTTV-3′ R1-N6 primer sequence (step 151 in the library prep protocol in [2])-5′TCGTCGGCAGCGTCAGATGTGTATAAGAGACAGNNNNNN-3′ PCR primer sequences (steps 157 and 160 in the library prep protocol in [2])-5′-AATGATACGGCGACCACCGAGATCTACACXXXXXXXXTCGTCGGCAGCGTC-3′, where XXXXXX is an index sequence for multiplexing libraries. 5′- CAAGCAGAAGACGGCATACGAGATGGGTGTCGGGTGCAG-3′. With these modifications in the primer sequences, custom sequencing primers are no longer required.

### scRNA-seq Data Quality Controls and Cell-type Cluster Identification

inDrops-seq raw data were analyzed and processed into transcript count matrix using the BradhamLab pipeline for inDrops scRNA-seq (https://github.com/BradhamLab/indrops).

The raw sequencing data were preprocessed to ensure high-quality data. Adapter sequences were trimmed, low-quality reads were filtered out, and potential contaminants were removed. After preprocessing, reads were aligned to the mouse transcriptome. The raw data from inDrops scRNA-seq included cell barcodes and unique molecular identifiers (UMI). The uniquely barcoded cells were separated, and the count of UMIs associated with each gene for each cell was determined.

For all analyses, the mouse genome (GRCm38) was adopted as reference. The resultant gene expression matrix was imported into R for further analysis. Cell filtering, data normalization, and clustering were performed utilizing the R package Seurat (36) version 4.3.0. The percentage of mitochondrial genes and the total number of expressed genes were calculated for each cell. Cells with a mitochondrial-to-endogenous gene expression ratio exceeding 0.2 were excluded as potential dying cells. In addition, cells expressing fewer than 200, or more than 8,000 total genes were discarded as potentially uninformative or multiplet cells. Counts were normalized using the Seurat function NormalizeData with default parameters. The expression data were then scaled using the ScaleData function, considering the number of unique molecular identifiers, the percentage of mitochondrial gene expression, and the difference between S and G_2_–M scores. Cell cycle scores were determined using the CellCycleScoring function.

Integration of various single-cell datasets into a unified object was achieved using the Harmony v1.0 approach from the R package (37) to address experimental and biological confounding factors and eliminate batch effects. Subsequently, dimensionality reduction was performed through principal component analysis on the batch-corrected data. Uniform Manifold Approximation and Projection (UMAP) dimensionality reduction (38) was adopted to the calculated principal components to obtain a two-dimensional representation for data visualization. Cell clusters were identified using the Louvain algorithm with a resolution of *r* = 1.2, implemented by the FindCluster function of Seurat. Comprehensive manual annotation was conducted to characterize each cluster. Different resolutions were used depending on the different cell types under investigation: *r* = 0.6 for epithelial cells and macrophages, and *r* = 1.2 for endothelial cells and fibroblasts.

Custom annotation was established to differentiate immune cells and nonimmune cells. The first annotation was performed using SingleR and ImmGen reference (39). The second annotation was carried out separately for the nonimmune cells (such as epithelial, stromal cells, and fibroblasts) using the Mouse Cell Atlas as reference (40).

Differential expression (marker) genes for the annotated clusters were identified using the Seurat package functions FindAllMarkers (to compare one cluster against all others iteratively) and FindMarkers (to compare two conditions) with default parameters. Significantly differentially expressed genes were determined on the basis of adjusted *P* values <0.05, average log fold-change >0.25, and a percentage of cells with expression >0.1.

Downstream analysis, including gene set enrichment analysis (GSEA), was conducted using the R/Bioconductor package ClusterProfiler with databases such as Gene Ontology (GO), Kyoto Encyclopedia of Genes and Genomes (KEGG) Pathway Database, Reactome Pathway Database, and Molecular Signatures Database. Enriched terms with a *q* value <0.05 were considered statistically significant. Heat maps were generated using the R package pheatmap, and charts and images were produced using the R package ggplot2.

### Trajectory Analysis

Trajectory analysis was performed using the python package STREAM (single-cell trajectories reconstruction, exploration, and mapping; 41). STREAM was used to reconstruct and visualize differentiation trajectories of epithelial cells based on the differential gene (markers) detected in the previous single-cell analysis.

### CellChat

Cell-cell communication was evaluated using the R package CellChat (v. 1.6.1; ref. 42). The mouse CellChatDB was used as reference.

### EnrichR Analysis

The top 300 enriched marker genes per cluster were submitted to the EnrichR enrichment suite (43, 44).

### Statistical Analysis and Reproducibility

For comparison of continuous variables between groups, we used *t* test (two-tailed; type 3) unless otherwise stated. Differences were considered statistically significant at *P* <0.05 (*), <0.01 (**), <0.001 (***; indicated by asterisks). The association between categorical variables was investigated with two-sided Fisher exact test on cell numbers per cluster. Statistical analyses were performed in GraphPad Prism 10 and R version 4.1.3 (The R Foundation for Statistical Computing) at 5% significance level. The specific details are provided in the text and in each figure legend.

### Data Availability

The datasets generated during and/or analyzed during the current study are available in the Gene Expression Omnibus repository: GSE253658. All other data are available from the corresponding author on reasonable request.

## Results

### BMI-1 Inhibition Affects Cell Cycle Progression and Tumor Growth

In previous studies, we showed that targeting BMI-1 with the preclinical compound PTC-209 (PTC Therapeutics) resulted in significant *in vivo* antitumor activity in xenograft lung cancer models characterized by low C/EBPα expression (25). We also demonstrated that the new clinical compound Unesbulin, identified by its ability to kill BMI-1^+^ cancer cells (24), that favorably completed phase I clinical trial in patients with advanced solid tumors (24), affects lung tumor progression in Kras-mutant/p53-null mice (23). Because mutations in the *EGFR* gene affect a vast percentage of patients with NSCLC (15), in this study we tested Unesbulin antitumor activity on the EGFR-mutant background.

**FIGURE 1:**
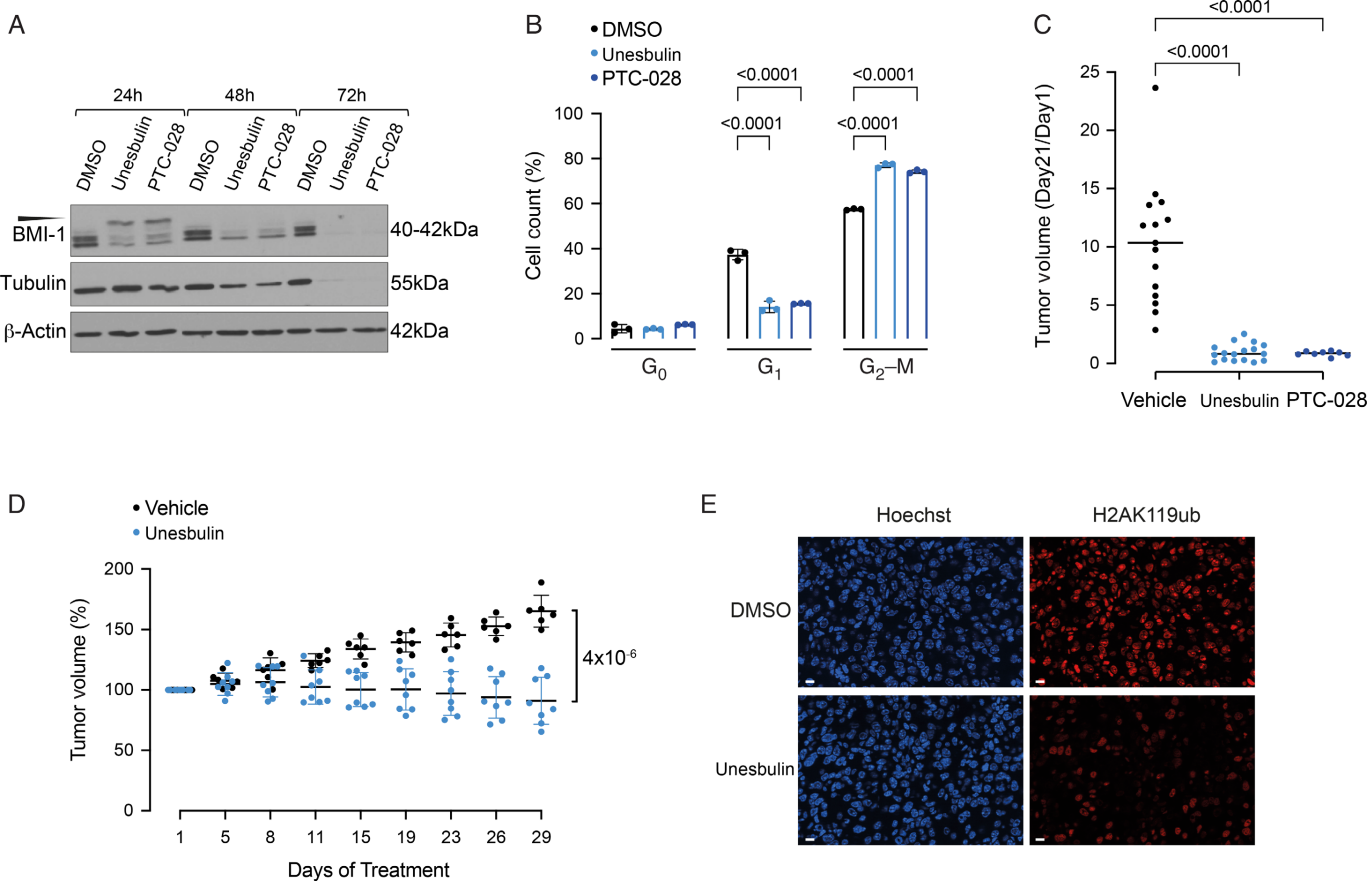
BMI-1 inhibition affects cell cycle progression and tumor growth. **A,** Western blot analyses of the human H1975 cell line treated for 24, 48, and 72 hours with DMSO Vehicle as control, and Unesbulin or PTC-028. Protein lysates were immunoblotted with an anti-BMI-1 or an anti-Tubulin antibody. Loading was assessed with an anti-β-actin antibody. Expected size is shown in kDa. The slower migrating hyperphosphorylated BMI-1 band is indicated by the arrowhead. **B,** Cell cycle analysis of the H1975 cell lines after treatment for 24 hours with DMSO (black), Unesbulin (light blue), or PTC-028 (dark blue). The bar charts represent the distribution of cells in G_0_, G_1_, and G_2_–M phases. *P* values are indicated. Error bars represent SD. Percentages of cells in each cell cycle phase are indicated. **C,** H1975 xenografts tumor volumes at treatment termination (Vehicle *n* = 15, black; Unesbulin *n* = 17, light blue; PTC208 *n* = 8, dark blue) normalized to the tumor volume measured at the beginning of treatment. The difference in tumor size at day 21 was statistically significant (*P* values are indicated). **D,** The graph shows percentage of change in transgenic mice tumor volume measured by MRI at the indicated timepoints, between Unesbulin (*n* = 8, light blue) and Vehicle-treated (*n* = 6, black) groups. Error bars represent SD. *P* value is indicated. **E,** Confocal microscopy fluorescence nuclear imaging of DNA and H2AK119ub in tumor tissues collected at the end of treatment. Top panels show a representative image of DMSO-treated tumor; bottom panels show a tumor treated with Unesbulin. Left panels show DNA staining by Hoechst 33342 (blue acquisition channel). Right panels show H2AK119ub staining by immunofluorescence (scale bar, 10 µm).

Therefore, as a first step, we performed *in culture* BMI-1 inhibition studies, using Unesbulin to assess its effects in culture on the human BMI-1^+^ NSCLC cell line H1975 (25), which carries T790M/L858R mutations in EGFR (25). We also adopted the drug analog PTC-028 to further corroborate our observations, because treatment with either Unesbulin or PTC-028 reportedly results in hyperphosphorylation of BMI-1 (24, 25, 45) and cell cycle arrest in G_2_–M phase (46). Immunoblotting with an anti-BMI-1 antibody the total cell lysates of H1975 cells undergoing treatment for 24, 48, and 72 hours using either Unesbulin or PTC-028 showed comparable hyperphosphorylation patterns for both compounds (Fig. 1A). At 24 hours, BMI-1 hyperphosphorylation reached its peak, followed by BMI-1 protein depletion at 48 and 72 hours, relative to 0.5% DMSO-treated cells. It has been recently reported that BMI-1 protein downregulation occurs in parallel to the potent induction of G_2_–M mitotic arrest caused by Unesbulin-induced inhibition of tubulin polymerization (47). Our Western blot analysis corroborated these findings and indeed showed progressive tubulin degradation at each timepoint following Unesbulin treatment as compared with DMSO (Fig. 1A).

To validate that BMI-1 activity was properly targeted, we determined whether levels of ubiquitinated Histone 2A (ubH2AK119) were affected by drug treatment, as ubiquitination of H2A represents the functional readout for BMI-1 activity (48, 49). BMI-1 interaction, through its own RING1 domain, with RING1B (one of the components of the PRC1 complex), enhances PRC1 E3 ligase activity, thus resulting in monoubiquitination of H2A on lysine 119 (48, 49). Notably, as shown by Western blot analysis, treating H1975 cells with Unesbulin for 24 hours significantly reduces ubH2AK119, as compared with DMSO-treated cells (Supplementary Fig. S1A), demonstrating Unesbulin affects BMI-1 function.

To further determine whether drug treatment affects cell cycle progression, H1975 cells were exposed to Unesbulin or PTC-028 for 24 hours, and cell cycle status was quantified using Pyronin Y and Vibrant Dye. Cell cycle analysis (Fig. 1B; Supplementary Fig. S1B) revealed G_2_–M arrest upon BMI-1 inhibition, with 80.7% (*P* < 1 × 10^−4^) and 77.3% (*P* < 1 × 10^−4^) cells in G_2_–M in Unesbulin- and PTC-028-treated cells, respectively, relative to 57.9% in DMSO-treated cells.

Having shown that anti-BMI-1 molecules affect in culture cell cycle progression, we next tested their effect on *in vivo* tumor growth, by generating xenograft models of H1975 in immunocompromised NSG mice. We started by subcutaneously injecting 1 × 10^6^ cells per flank and let the tumors establish to approximately 100 mm^3^ size; thereafter, mice were treated twice a week with Unesbulin (*n* = 17), PTC-028 (*n* = 8), or Vehicle (*n* = 15) for 28 days. As shown (Fig. 1C), the average tumor volume at treatment termination, normalized to day 0, was found to be significantly decreased by 89.93% (*P* = 1.49 × 10^−5^) and 91.82% (*P* = 1.21 × 10^−5^), in individually-measured tumors obtained from mice receiving Unesbulin or PTC-028, relative to Vehicle-treated tumors, respectively. To assess whether *in vivo* antitumor effects were obtained via BMI-1 downregulation, we also performed BMI-1 genetic knockdown using shRNA. In detail, H1975 cells were transduced with two different GFP-coupled lentiviral shRNAs against BMI-1 (sh1-BMI-1 and sh2-BMI-1) or a control shRNA (sh-luciferase). GFP^+^ cells were sorted and BMI-1 knockdown confirmed by Western blot analysis (Supplementary Fig. S1C). Subsequently, 270,000 cells per construct were subcutaneously implanted into NSG mice [sh1-BMI-1 (*n* = 1); sh2-BMI-1 (*n* = 2); and sh-luciferase (*n* = 1); one flank per construct]. Tumor growth was negatively affected up to 17 days in mice injected with sh1-BMI-1– or sh2-BMI-1–, as compared with sh-luciferase–infected cells (Supplementary Fig. S1D), resulting in formation of smaller tumors versus control (Supplementary Fig. S1E). Overall, these data show that *BMI-1* knockdown in H1975 cells affects tumorigenesis similarly to Unesbulin-mediated and PTC-028–mediated effects, supporting the hypothesis that BMI-1 pharmacologic inhibition achieved through these novel compounds inhibits tumor growth in a BMI-1–dependent manner.

Having demonstrated that BMI-1 activity plays a critical role in supporting the EGFR^TL^ tumorigenic phenotype, we then selected the clinical grade Unesbulin drug to evaluate BMI-1 through *in vivo* inhibitory strategies. To this aim, because IHC analysis of ADCs growing in EGFR^TL^ transgenic mice proved high positivity for BMI-1 expression, as compared with healthy controls (Supplementary Fig. S1F), we adopted this model for our validation studies. To investigate whether pharmacologic treatment with Unesbulin was sufficient to reduce tumor burden, doxycycline-inducible EGFR^TL^ mice were placed on doxycycline and monitored by MRI for tumor growth, as reported previously (28–30). Treatment with Unesbulin (or Vehicle control) was randomized when tumor size reached approximately 100 mm^3^. Mice received Unesbulin (*n* = 8) or Vehicle (*n* = 6), twice a week, as performed previously (23). Changes in tumor volume over the course of treatment for 4 weeks were calculated as percent change in volume over tumor volume at day 1 of treatment, which was set as 100% (23, 28–30). As shown in Fig. 1D, at treatment termination, tumor volume of Vehicle-treated mice increased by 165.2% ± 13.21%, while Unesbulin-treated tumors shrank to 91.1%±19.52%, resulting in a significant overall tumor-growth ability reduction of 55.1% (*P* = 4 × 10^−6^). Immunoblotting of tumors at 4-week treatment showed major reduction in ubH2AK119 (Supplementary Fig. S1G) in Unesbulin- versus DMSO-treated tumors, demonstrating *in vivo* functional reduction of BMI-1 activity. Similarly, immunofluorescence staining revealed strong nuclear staining of ubH2AK119 that colocalizes with Hoechst chromatin staining in control-treated tumors, whereas treatment with Unesbulin significantly reduced the number of ubH2AK119 positive *foci* (Fig. 1E), further confirming BMI-1 activity is being targeted via Unesbulin, resulting in *in vivo* tumor shrinkage.

### scRNA-seq Reveals Pulmonary Milieu Clusters in Healthy and Tumor-bearing Lungs

Lung cancer milieus consist of numerous distinct subpopulations. Thus, studying bulk cell populations provides only limited knowledge of biological systems, while interrogation of individual cells provides deeper insights into processes masked at the population level (50). To identify the different subpopulations in the TME, as well as investigate how they are transcriptionally altered at the single-cell level after drug treatment, we performed scRNA-seq on both EGFR^TL^ tumors from mice treated with Unesbulin or Vehicle for 1 month, and compared them with healthy lungs. This approach revealed the diversity and dynamics of individual cells in the TME. We obtained 41 transcriptional clusters (C0–C40) from freshly isolated tissues of untreated and Vehicle-treated healthy lungs (Supplementary Fig. S2A). Cluster quantification (Supplementary Fig. S2B) demonstrated that Vehicle treatment caused only minor changes in cell percentages (Supplementary Table S1), that consequently we adopted as our reference control (from now on referred to as healthy lungs). We then compared freshly isolated tissues from healthy lungs (3,952 cells) obtained from control littermates, with EGFR^TL^ tumors after 1 month of Vehicle (11,000 cells) or Unesbulin (6,889 cells) treatment. We used the healthy lung clusters as a baseline to identify the transformed cells in the tumor tissues, and characterize them as either tumor-specific or tumor-enriched.

**FIGURE 2:**
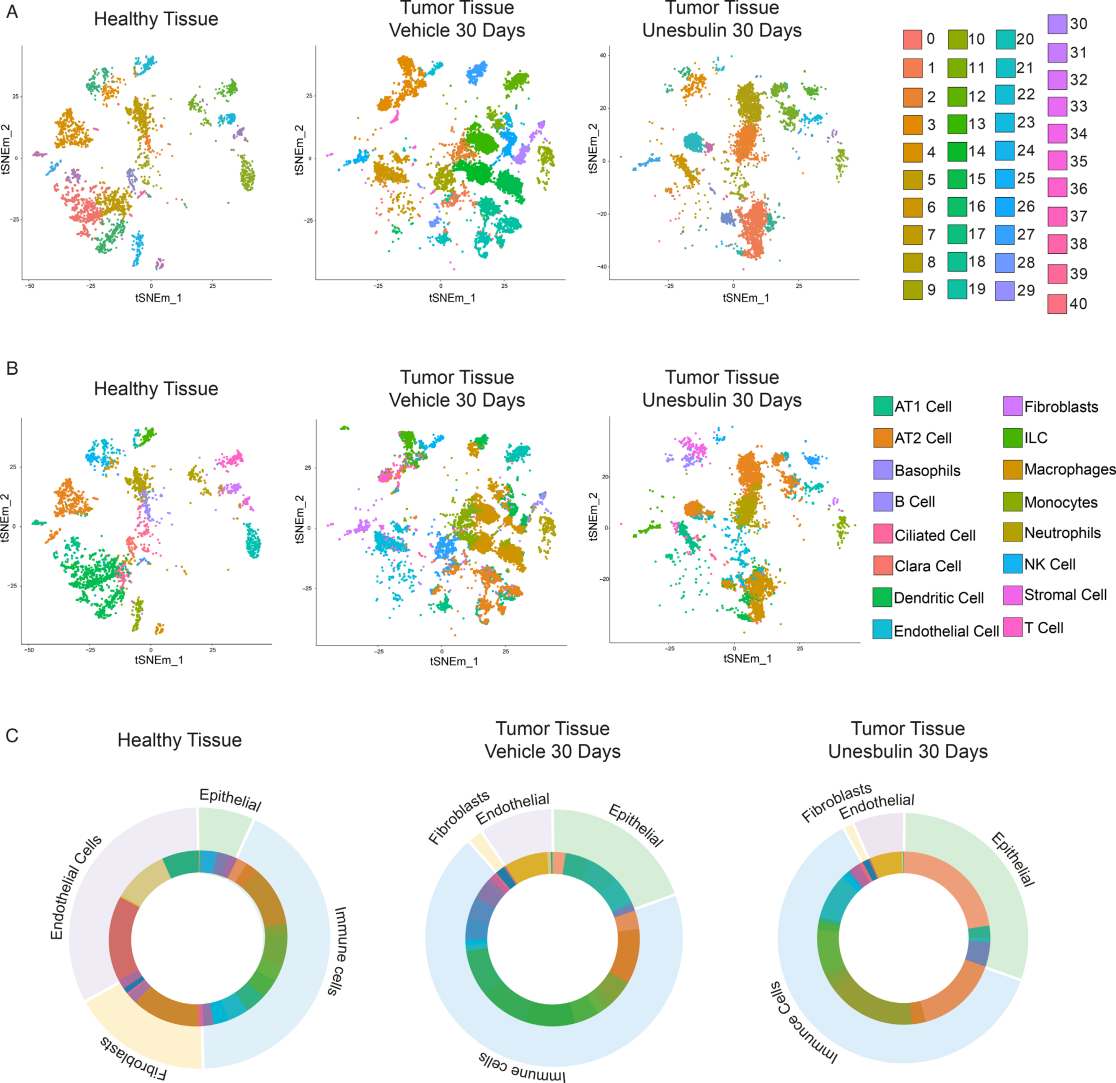
scRNA-seq of healthy and tumor-bearing lungs highlights presence of pulmonary milieu clusters. **A,** t-SNE plot of the 41 clusters identified in healthy lungs (*n* = 3, left), and Vehicle- (*n* = 10, middle), or Unesbulin-treated tumors (*n* = 3, right), at 30 days. Each point represents one cell. Each color represents a defined transcriptional cluster as shown in the corresponding legends. **B,** t-SNE plot in which transcriptional clusters have been annotated as epithelial, immune, endothelial, or fibroblast subpopulations. **C,** Cluster distribution superimposed to clusters annotation within epithelial, immune, endothelial cells, and fibroblasts in healthy lungs (left), and Vehicle- (middle), or Unesbulin-treated tumors (right).

Samples were pooled, batch-corrected, clustered, and visualized using t-distributed stochastic neighbor embedding (t-SNE) plots. We detected 41 t-SNE clusters (Fig. 2A; Supplementary Tables S2 and S3) and identified specific cell subpopulations in the healthy or the tumor samples (Fig. 2B; Supplementary Tables S4 and S5), by superimposing cell annotations on cluster distributions, as per Maroni and colleagues (23).

The dataset displayed four major subgroups: epithelial, immune, endothelial cells, and fibroblasts (Fig. 2C). Both the epithelial [alveolar type 1 (AT1), AT2, ciliated, Clara cells] and immune (basophils, B cells, dendritic cells, innate lymphoid cells, macrophages, mast cells, monocytes, neutrophils, natural killer, T cells) components were significantly enriched in the tumor samples (19.38% and 69.95%, respectively) compared to healthy lungs (9.31% and 43.61%, respectively; *P* < 1 × 10^−4^ in both cases), whereas endothelial and fibroblast cell percentages were lower in tumors (8.47% and 2.87%) compared with healthy samples (27.23% and 13.69%; *P* < 1 × 10^−4^ in both cases; Supplementary Table S4). Upon Unesbulin treatment epithelial cells shifted to 26.74%, immune cells to 63.47%, endothelial cells to 6.34% and fibroblasts to 2.45% (Supplementary Table S5). Each cellular subgroup included distinct t-SNE clusters (Fig. 2C), whose distribution varied among the healthy, Vehicle- and Unesbulin-treated tumor samples (Supplementary Tables S2 and S3).

### Deconvolution of Epithelial Clusters Highlights the Cancer Stem Cell Nature of the AT2-like Clusters C0 and C4 (C0^epi^ and C4^epi^)

**FIGURE 3:**
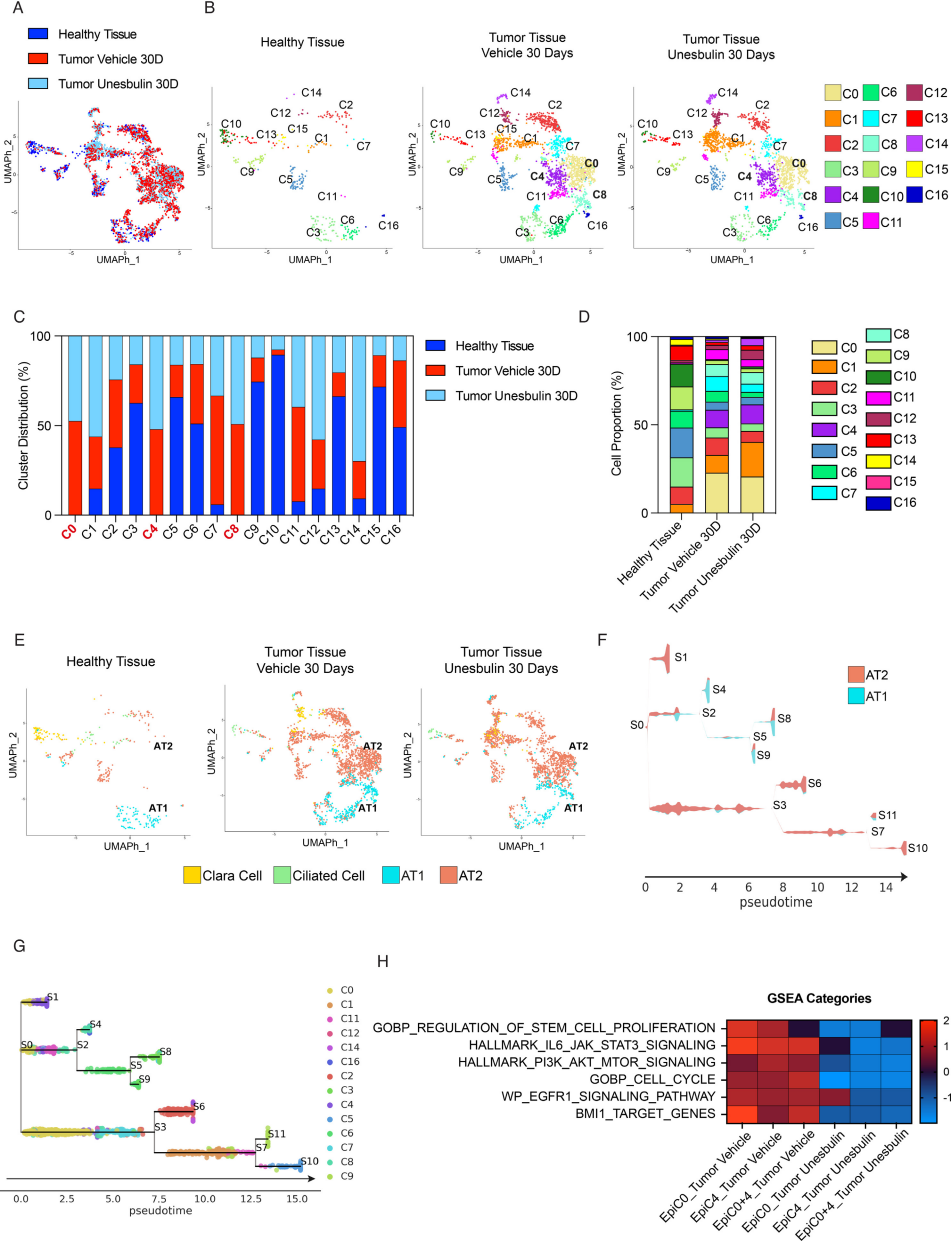
Deconvolution of healthy and diseased epithelial clusters highlights the malignant stem cell nature of C0^epi^ and C4^epi^. **A,** UMAP cluster distribution of epithelial cells from healthy lungs (*n* = 3, dark blue), Vehicle- (*n* = 10, red), and Unesbulin-treated tumors (*n* = 3, light blue), up to 30 days. **B,** Split distribution of the 17 epithelial transcriptional clusters (C0–C16) identified in healthy lungs (left), Vehicle- (middle), and Unesbulin-treated tumors (right). Each color represents a defined transcriptomic cluster. **C,** Histograms representing C0–C16 percentage distribution per sample (healthy lungs, Vehicle-, and Unesbulin-treated tumors). **D,** Histograms representing the percentage contributions of C0–C16 clusters per sample (healthy lungs, Vehicle-, and Unesbulin-treated tumors). **E,** UMAP cluster annotation of epithelial cells from healthy lungs, and Vehicle- and Unesbulin-treated tumors. Each color represents a different epithelial subpopulation. **F,** STREAM analysis on the AT2/AT1 subpopulations identifies states S0–S11. **G,** STREAM analysis on the AT2/AT1-annotating clusters depicts trajectories of differentiation. **H,** Heat map showing GSEA of Vehicle- and Unesbulin-treated C0^epi^-C4^epi^ identifies signaling pathways enriched in each sample and affected upon treatment.

ADCs are solid tumors that originate from alveolar cells, yet the epithelial compartment of these tumors remains underinvestigated, particularly at the single-cell resolution level. To address this gap, we performed UMAP dimensionality reduction and clustering on cells annotating as epithelial-like cells, obtaining a newly refined cluster distribution and improved resolution of the epithelial compartment. This approach revealed 17 epithelial clusters (C0-C16^epi^) based on the Louvain community detection algorithm. These clusters showed different patterns of distribution across healthy lungs and tumors treated with Vehicle or Unesbulin (Fig. 3A and B). To better compare the cluster sizes and composition, we separated the cells by sample type (Fig. 3B). The heat maps provided in Supplementary Fig. S3A show that each cluster has a specific and distinct transcriptional profile.

C1^epi^ and C11^epi^ are enriched in the tumor Vehicle samples (C1^epi^ = 10.18%, *P* = 1.9 × 10^−3^; C11^epi^ = 5.63%, *P* = 6 × 10^−6^) compared with the healthy tissue (C1^epi^ = 5.16%; C11^epi^ = 0.82%; Fig. 3C and D; Supplementary Table S6). C0^epi^, C4^epi^, and C8^epi^ are instead tumor-specific clusters (C0^epi^ = 22.80%, *P* < 1 × 10^−4^; C4^epi^ = 9.94%, *P* < 1 × 10^−4^; C8^epi^ = 6.85%, *P* = 6 × 10^−11^), being undetectable in healthy specimens (Fig. 3C and D; Supplementary Table S6).

On the other hand, C3^epi^, C5^epi^, C6^epi^, C7^epi^, C9^epi^, C10^epi^, C13^epi^, and C15^epi^ are more abundant in the healthy sample than in tumor-Vehicle samples (Fig. 3C and D; Supplementary Table S6). C2^epi^, C12^epi^, C14^epi^, and C16^epi^ have similar representation in both samples (Fig. 3C and D; Supplementary Table S6). The percentages and *P* values for each cluster are given in the figure legend.

C0^epi^, C4^epi^, and C8^epi^ were exclusively detected in the tumor tissue, suggesting they contain *bona fide* malignant cells (Fig. 3C and D; Supplementary Table S6). Among the tumor-enriched clusters, Unesbulin slightly reduced the size of C11^epi^, compared with Vehicle (4.23% vs. 5.63%; *P* = 4.83 × 10^−2^; Fig. 3C and D; Supplementary Table S7), while C1^epi^ was significantly enriched (C1^epi^ = 19.60% vs. 10.18%; *P* < 1 × 10^−4^). Conversely, the tumor-specific clusters C0^epi^, C4^epi^ and C8^epi^ remained sizewise unaffected by treatment (C0^epi^ = 20.63%, *P* = ns; C4^epi^ = 10.75%, *P* = ns; C8^epi^ = 6.62%, *P* = ns; Fig. 3C and D; Supplementary Table S7).

To highlight the features of each epithelial cluster, we superimposed the cell annotations obtained from the Mouse Cell Atlas (ref. 40; Fig. 3E) on the cluster distribution (Fig. 3B), and classified each cluster as a defined epithelial cell subtype. We found that AT1 cells are predominantly contained in four clusters: C3^epi^, C6^epi^, C8^epi^, and C16^epi^. Ciliated and clara cells mainly reside in C10-C13^epi^ and C15^epi^, respectively (Fig. 3E). AT2 cells are distributed in multiple clusters (C0^epi^, C1^epi^, C2^epi^ C4^epi^, C5^epi^, C7^epi^, C9^epi^, C11^epi^, C12^epi^, and C14^epi^), of which, C5^epi^, C7^epi^, and C9^epi^ (Fig. 3E) are mainly present in the healthy lung, thus representing normal-like AT2 cell clusters (Supplementary Table S8; Supplementary Fig. S3B). Supplementary Table S9 shows how the tumor subpopulations AT1 and AT2 are minimally affected upon Unesbulin treament.

To understand why the AT2-like tumor-exclusive clusters C0^epi^ and C4^epi^ failed to respond to Unesbulin, we explored the possibility that they had stem cell–like properties that hindered their sensitivity to treatment. Therefore, we performed STREAM analysis to reconstruct their hierarchy and disentangle the complex branching trajectories of AT1 and AT2 cell compartments within the tumor (Fig 3F). Using the AT1 and AT2 clusters as input, STREAM data revealed that the two initial branching states, S0 and S1, correspond indeed to AT2-like C0^epi^ and C4^epi^, respectively (Fig. 3G). This suggested that C0^epi^ and C4^epi^ may overall represent a cancer stem cell (CSC) tumor-initiating compartment that acts as early malignant module, that we named STEMMED (STem cEll tuMor-initiating coMpartment Early-malignant moDule). By comparing the molecular features and pathways of STEMMED versus the other tumor epithelial clusters, GSEA identified categories that were significantly activated in the module. Both the GO category “Stem Cell Proliferation” (Fig. 3H) and pathways involved in cell survival and proliferation (i.e., PI3K/AKT, mTOR, JAK/STAT), frequently switched on in tumors, were predicted to be activated in STEMMED (Fig. 3H). These data show that C0^epi^ and C4^epi^ are characterized by expression of distinct molecular mediators of tumor proliferation and growth, supporting our hypothesis that STEMMED contains actively proliferating malignant cells. Upon drug treatment such protumorigenic activities were indeed inhibited (Fig. 3H). We also performed GSEA using “Bmi-1 targets” as a category, because *Bmi-1* is a pivotal gene in CSCs, and we found that it was positively enriched in STEMMED versus the other tumor epithelial clusters. Upon exposure to Unesbulin, that affects Bmi-1 activity (24), this category shifted to being negatively enriched (Fig. 3H) in STEMMED, concomitantly to the cell cycle–related GO category (Fig. 3H). Interestingly, we observed that the activation of EGFR signaling was reduced by Unesbulin treatment in C4^epi^ and in the combination of both STEMMED clusters (Fig. 3H). However, EGFR signaling was not affected in C0^epi^ alone. In STREAM, we identified a cluster (S0 containing C0^epi^) that is exclusive to the tumor and has high EGFR signaling activity, consistent with the continuous exposure of the mouse model to doxycycline. We inferred that this cluster is higher in the malignant hierarchy within STEMMED. C0^epi^ contains CSCs with sustained EGFR signaling activity, thus representing a tumor-initiating cluster, that we named START (Stem Tumor initiAtor egfR clusTer).

Taken together, our results demonstrate that Unesbulin treatment affects key molecular pathways that support tumor proliferation and survival *in vivo*. This is especially true for the malignant AT2-like STEMMED module, in line with the MRI data on tumor cell growth arrest after 30 days of drug treatment *in vivo* (Fig. 1D).

### Deconvolution of Macrophage Clusters Uncovers Interstitial and Alveolar Subpopulations and Their Cross-talk With START

**FIGURE 4:**
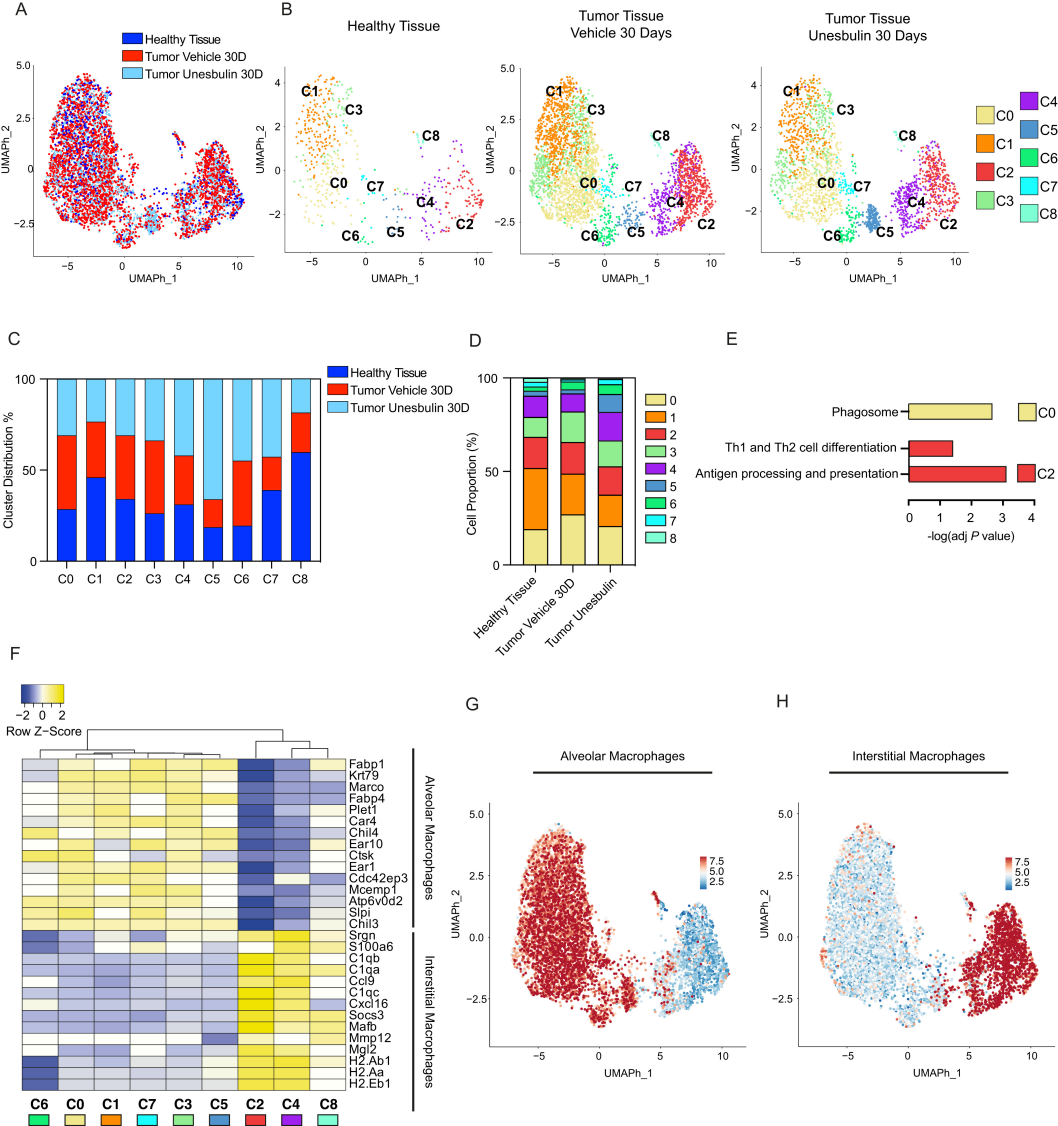

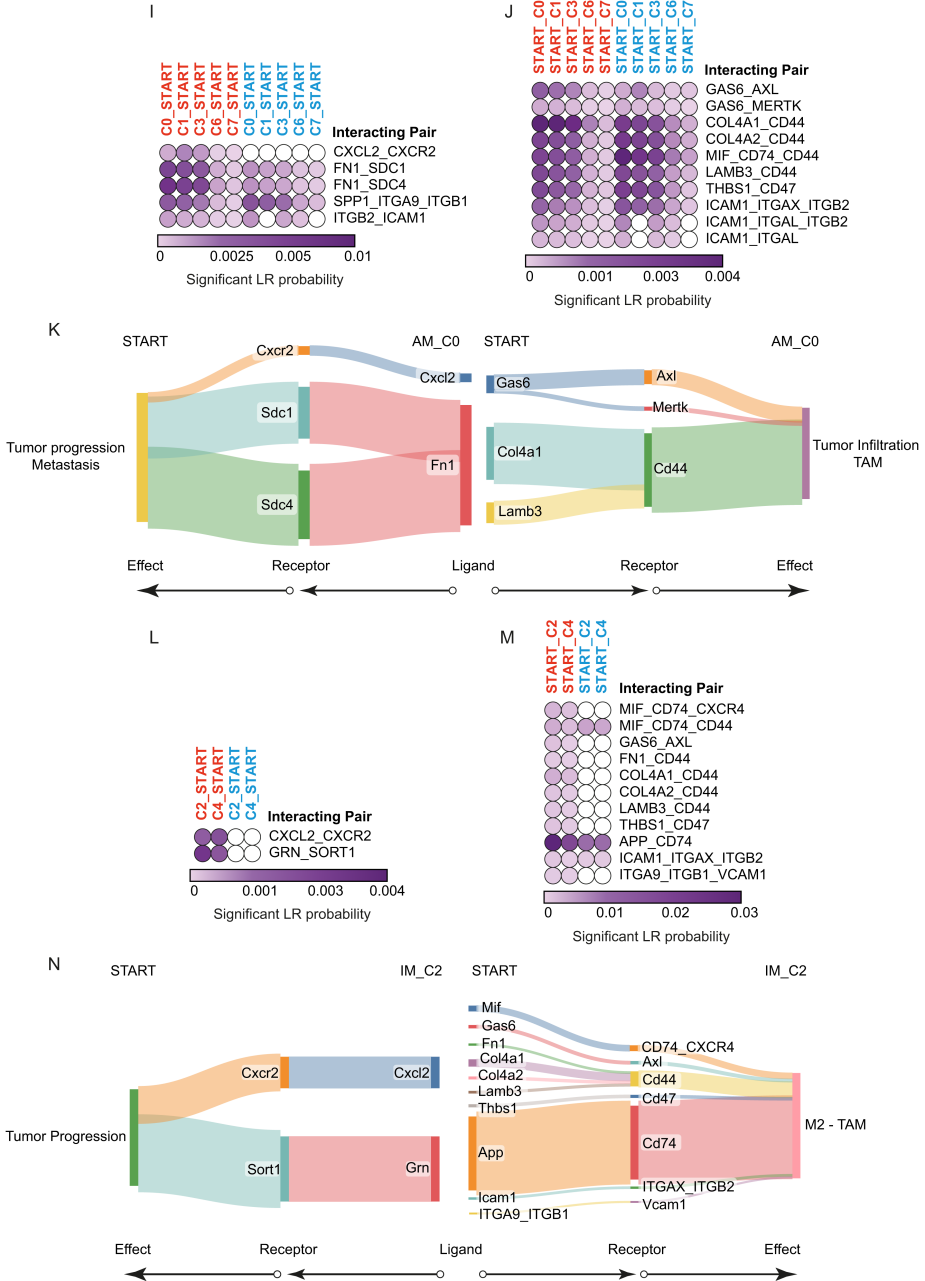
Deconvolution of healthy and diseased macrophage clusters demonstrates presence of interstitial and alveolar subpopulations and identifies tumor-specific cross-talks occurring in the malignant stem epithelial compartment. **A,** UMAP cluster distribution of macrophages from healthy lungs (*n* = 3, dark blue), Vehicle- (*n* = 10, red), and Unesbulin-treated tumors (*n* = 3, light blue), up to 30 days. **B,** Split UMAP distribution of the nine macrophage transcriptional clusters (C0–C8) identified in healthy lungs (left), Vehicle- (middle), and Unesbulin-treated tumors (right). Each color represents a defined transcriptional cluster. **C,** Histograms representing macrophage cluster percentage distribution per sample (healthy lungs in blue, Vehicle- and Unesbulin-treated tumors in red and light blue, respectively). **D,** Histograms representing the percentages contribution of macrophage clusters per sample (healthy lungs, Vehicle- and Unesbulin-treated tumors). **E,** KEGG analysis of identified signaling pathways differentially enriched in selected macrophage clusters (C0 and C2). **F,** Heat map showing AM and IM expressed genes in healthy macrophage clusters. UMAP plot representing the expression of the representative category-specific signature in AM (**G**) and IM (**H)**. **I** and **J,** Heat maps showing the predicted interactions between AM clusters and START in Vehicle- (red) and Unesbulin-treated tumors (light blue). **K,** Sankey plot of the protumorigenic phenotypes promoted on START by alveolar C0 macrophage-secreted ligands (left) and on macrophages by START secreted ligands (right). **L** and **M,** Heat maps showing the predicted interactions between IM clusters and START in Vehicle- (red) and Unesbulin-treated tumors (light blue). **N,** Sankey plot of the protumorigenic phenotypes promoted on START by C2 IM-secreted ligands (left) and on macrophages by START secreted ligands (right).

Because macrophages were the most abundant subtype of tumor-infiltrating immune cells, we visualized their transcriptome separately from those of other hematopoietic cells (Supplementary Table S4). UMAP representation (Fig. 4A) revealed an overall overlap of healthy and tumor samples. Macrophage cluster distribution identified nine sets (C0–C8; Fig. 4B) that show a specific molecular signature, as illustrated in the heat maps of Supplementary Fig. S4A. All clusters were represented in the healthy lungs, as well as both tumor tissues (Vehicle- and Unesbulin-treated), but at different percentages (Fig. 4C). Our data show that the transcriptome of macrophages in healthy and tumor tissues is heterogeneous and dynamic (Supplementary Table S10), and that Unesbulin treatment affects the percentage of some macrophage clusters (Supplementary Table S11). In details, cluster quantification showed that there was no significant difference in the percentages of C2^macro^, C4^macro^, and C5^macro^ between healthy lungs and sham-treated tumors (Fig. 4D). In contrast, C1^macro^, C7^macro^, and C8^macro^ were enriched in healthy lungs, compared to tumors (C1^macro^: 32.70% vs. 21.68%, *P* < 1 × 10^−4^; C7^macro^: 2.49% vs. 1.16%, *P* = 2.24 × 10^−2^; C8^macro^: 2.10% vs. 0.76%, *P* = 1.17 × 10^−2^). C0^macro^ was instead 1.4-fold higher in tumors, than healthy lungs (27.08% vs. 19.12%; *P* = 9 × 10^−5^). Similarly, C3^macro^ is 1.58-fold enriched in tumors (16.89% vs. 10.71%; *P* = 2.10^−4^) and C6^macro^ is 1.83-fold enriched in tumors versus normal lung (4.18% vs. 2.29%; *P* = 3.88 × 10^−2^).

Unesbulin treatment affected the percentage of some macrophage clusters in different ways (Supplementary Table S11). While the drug treatment did not change the percentage of C2^macro^, C6^macro^, and C8^macro^, it increased the percentage of C4^macro^ (15.27% vs. 9.68%, *P* = 8 × 10^−10^), C5^macro^ (9.53% vs. 2.20%, *P* < 1 × 10^−4^), and C7^macro^ (2.73% vs. 1.16%, *P* = 3 × 10^−5^), in tumor tissues (Fig. 4D). On the other hand, Unesbulin treatment decreased the percentage of C0^macro^ (20.82% vs. 27.08%, *P* = 2 × 10^−7^) and C1^macro^ (16.75% vs. 21.68%, *P* = 2 × 10^−6^), and slightly reduced the percentage of C3^macro^ (13.84% vs. 16.27%, *P* = 1.52 × 10^−2^) in tumor tissues.

To infer the function of each transcriptionally-defined clusters, we performed a pathway analysis (KEGG), using the differentially expressed genes per cluster list from the healthy dataset. The clusters with the most significant differences were C0^macro^ and C2^macro^. C0^macro^ showed activation in the Phagosome Maturation category and C2^macro^ was enriched in the Antigen presentation and Th2 pathways (Fig. 4E), suggesting possible interactions with other immune cells. These categories are consistent with the known functions of the two main subtypes of macrophages present in the lung: alveolar (AM) and interstitial macrophages (IM), respectively.

After characterizing the molecular signature of each cluster, we used it to classify them into different macrophage subtypes. We compared a specific signature capable of defining AMs and IMs, and generated the heat map shown in Fig. 4F, in which the dendrogram identifies the two distinct subgroups of macrophages. The first branch consists of C6^macro^, C0^macro^, C1^macro^, C7^macro^, C3^macro^, and C5^macro^, which belong to the tissue-resident AMs category, based on the known markers. The second branch consists of C2^macro^, C4^macro^, and C8^macro^ which are IMs. Finally, we observed that C5^macro^, although belonging to the AM branch in the healthy sample, did not show strong AM marker enrichment in the tumor tissue and shifted identity toward the IM branch in the tumor (Supplementary Fig. S4B and S4C). Similarly, C8^macro^ did not show a strong IM signature in tumors, whether they were treated with Vehicle or Unesbulin (Supplementary Fig. S4B and S4C).

UMAP plots in Fig. 4G and H show the expression of these category-specific signatures adopted to classify the macrophages into AM and IM subgroups. Both plots clearly display that each subgroup clusters together, indicative of their distinct molecular profile. To dig further into their functionality and identify the molecular axes that specifically characterize their cross-talk with START, we used CellChat to predict the cellular receptor–ligand interactions in both Vehicle- and Unesbulin-treated tumors. Specifically, we investigated the interactions between AM and IM clusters with START. Our data revealed that all the AM clusters adopt the same interaction pairs with START (Fig. 4I and J). The heat maps also show that almost all the interactions are weakened or vanish upon Unesbulin treatment. Moreover, when assessing the tumor-specific axes between AM and START that are affected by Unesbulin treatment, our interactome data support the hypothesis that ligands secreted by START, such as GAS6, COL4a1, and LAMB3, induce a protumor phenotype acquisition [tumor-associated macrophage (TAM) phenotype] in the macrophages that express their cognate receptors (Fig. 4K). Concomitantly, ligands secreted by the AM macrophages, such as FN1, stimulate tumor growth by signaling via the START cell surface receptors (Fig. 4K). When observing the interactions between IM and START, the heat maps highlight that almost all the interactions are disrupted or diminished by Unesbulin (Fig. 4L and M). Ligands secreted by START, such as GAS6 and ICAM, lead to a protumoral phenotype acquisition (M2 phenotype) in the IMs, while CXCL2 and GRN, secreted by IMs, trigger tumor growth in START (Fig. 4N).

### Comparison of Endothelial Cells and Fibroblasts from Healthy Lungs and Tumors Characterize Their Distribution and Tumor-specific Interplay with START

**FIGURE 5:**
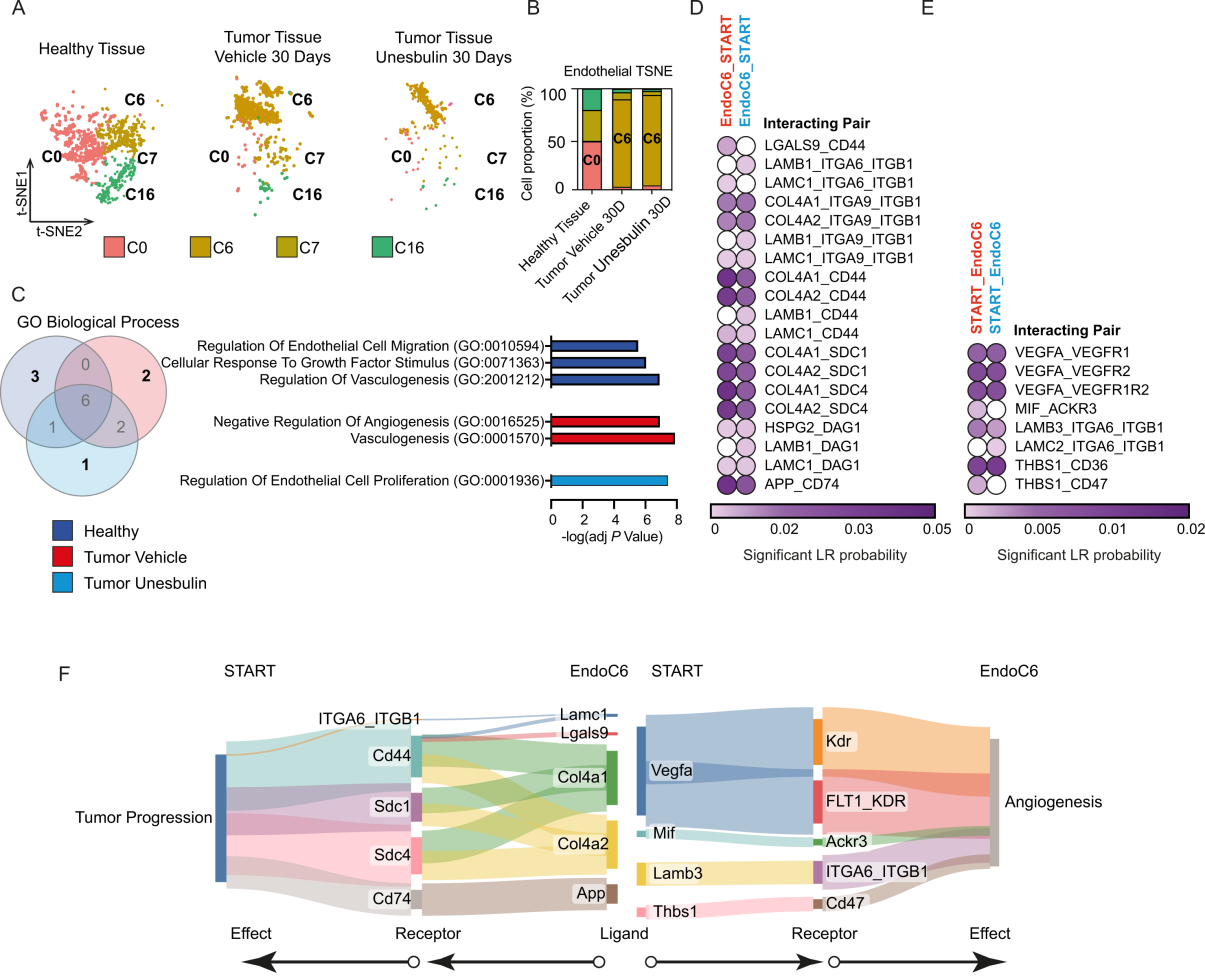

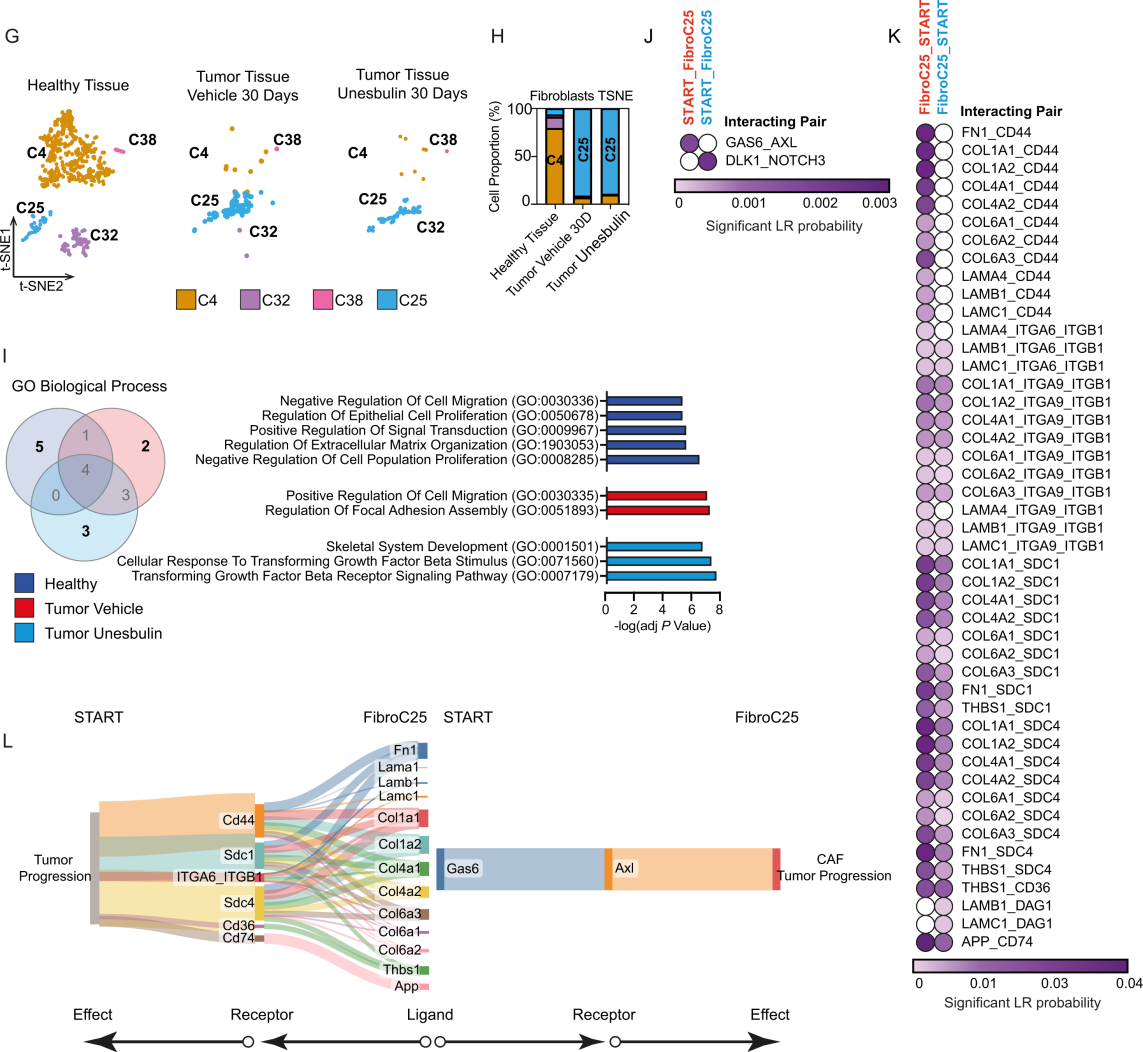
Comparison of pulmonary healthy and diseased endothelial cells and fibroblasts characterize their distribution and tumor-specific interplays with START. **A,** Split t-SNE plot distribution of the four endothelial clusters identified in healthy lungs (left), Vehicle- (middle), and Unesbulin-treated tumors (right). Each color represents a defined transcriptional cluster. **B,** Histograms representing the percentage contribution of endothelial clusters per sample (healthy lungs, Vehicle- and Unesbulin-treated tumors). **C,** Venn diagram showing common and unique GO categories/ biological processes enriched in endothelial cells from each sample (healthy lungs, Vehicle- and Unesbulin-treated tumors). **D,** Heat map showing the predicted interactions between endothelial cells and START in Vehicle- (red) and Unesbulin-treated tumors (light blue). **E,** Heat map showing the predicted interactions between START and endothelial in Vehicle- (red) and Unesbulin-treated tumors (light blue). **F,** Sankey plot of the protumorigenic phenotypes promoted on START by endothelial-secreted ligands (left) and on endothelial cells by START-secreted ligands (right). **G,** Split t-SNE plot distribution of the fibroblast clusters identified in healthy lungs (left), Vehicle- (middle), and Unesbulin-treated tumors (right). Each color represents a defined transcriptional cluster. **H,** Histograms representing the percentage contribution of fibroblast clusters per sample (healthy lungs, Vehicle-, and Unesbulin-treated tumors). **I,** Venn diagram showing common and unique GO categories/biological processes enriched in fibroblasts from each sample (healthy lungs, Vehicle- and Unesbulin-treated tumors). **J,** Heat map showing the predicted interactions between START and fibroblasts in Vehicle- (red) and Unesbulin-treated tumors (light blue). **K,** Heat map showing the predicted interactions between fibroblasts and START in Vehicle- (red) and Unesbulin-treated tumors (light blue). **L,** Sankey plot of the protumorigenic phenotypes promoted on START by fibroblast-secreted ligands (left) and on fibroblasts by START-secreted ligands (right).

Using scRNA-seq analysis, we characterized both healthy and tumor-associated endothelial cells and fibroblasts, in addition to epithelial and immune cells. By superimposing the cluster distribution (Fig. 2A) on the cell annotation (Fig. 2B), we partitioned these populations into more refined transcriptional entities. Specifically, in the endothelial compartment, the most abundant clusters within the healthy tissue are C0^endo^, C7^endo^, and C16^endo^, representing the vast majority of healthy endothelial cells (C0^endo^:47.95%; C7^endo^: 30.66%; C16^endo^: 20.54%; Supplementary Table S12). Conversely, most of the tumor endothelial cells fall into C6^endo^ (86.59% and 89.59% in Vehicle- and Unesbulin-treated tumors, respectively), which is instead barely detectable in healthy tissues (Fig. 5A and B; Supplementary Tables S12 and S13), that therefore represents a tumor-enriched cluster. To further characterize the transcriptional differences between the clusters, we performed GO analysis using EnrichR on the significant upregulated genes within each sample. Figure 5C shows the Venn diagram of the top 10 statistically significant GO Biological Process terms enriched in each sample, displaying the common and unique categories. Vehicle-treated tumors uniquely show enrichment of two categories (“negative regulation of angiogenesis” and “vasculogenesis”), indicative a possible hypoxic environment that may function as a stimulus to increase vasculogenesis. However, upon Unesbulin treatment, the category of “regulation of endothelial cell proliferation” is instead enriched. Furthermore, CellChat interactome analysis revealed specific interactions between tumor endothelial cells and START, which are decreased or disrupted upon Unesbulin treatment (Fig. 5D and E). Figure 5F shows the tumor specific axes, with the Col4a1/2_SCD system and the VEGF signaling pathway being the most represented. Our analysis show that their downstream effects are context dependent, in that they lead to increased proliferation and metastasis formation in tumor cells but lead to increased angiogenesis in endothelial cells (Fig. 5F).

Healthy and tumor-associated fibroblasts were distributed across different clusters (Supplementary Table S14). We discovered that while healthy fibroblasts are predominantly confined in C4^fibro^ (89.76%), the majority of tumor-associated fibroblasts are mainly contained in C25^fibro^ that represents 93.60% and 90.32% of Vehicle- and Unesbulin-treated tumor cells, respectively (Fig. 5G and H; Supplementary Table S15). Figure 5I shows the Venn overlap of the most significant GO biological processes enriched in Vehicle- and Unesbulin-treated tumors. The sham-treated tumors showed enrichment for two GO categories: “Positive regulation of cell migration” and “Regulation of focal adhesion assembly”, suggesting their proinvasive nature. The Unesbulin-treated tumors did not show enrichment for these categories. Instead, they were characterized by enrichment for three GO categories that were unique to the drug-treated set: “Skeletal system development,” “Cellular response to TGFb stimulus,” and “Transforming TGFb signaling pathway.” These categories may suggest a different response to growth factors. Moreover, CellChat interactome analysis highlighted that fibroblasts (C25^fibro^) contained in the sham-treated tumor establish an intense cross-talk with START (Fig. 5J and K), mainly based on Col4/6_SDC and CD44. Figure 5L shows the effects of ligands secreted by malignant AT2 cells to hijack the cancer-associated fibroblasts (CAF). Once signals are internalized by the fibroblasts, they cause deregulation of extracellular matrix (ECM) deposition. This signaling pathway leads to tumor growth and metastasis in Vehicle-treated tumors, and Unesbulin treatment disrupt these protumorigenic axes (Fig. 5L).

## Discussion

NSCLC is the single largest contributor to cancer-related mortality worldwide (51). EGFR mutations are common in NSCLC with ADC histology (52). However, the development of targeted therapies for EGFR-mutant ADC is hindered by tumor heterogeneity and complex TME interactions that influence tumor progression and treatment responses (53, 54). The complexity of the epithelial component of solid tumors, and its intermingled microenvironment, which is symbiotic with cancer cells (55), is still underinvestigated, making it difficult to identify better targeting strategies. Therefore, high-resolution mapping of the epithelial and the plethora of cells populating the TME subtending mutant EGFR-driven ADC growth is a critical gap to fill. This endeavor sets the basis to identify approaches to target cancer epithelial cells and modulate the cross-talk with the TME, which is responsible for supporting tumor growth.

Our previous data on the single-cell transcriptomics of mutant KRAS/p53-null lung ADCs and healthy lungs, identified a unique tumor epithelial cluster/subpopulation (23). This population exhibits a conserved signature when compared with a distinctive cluster that is only present in clinical KRAS-mutant ADCs, showing exceptional parallelisms across species. In addition, our data also show dynamic changes in this transformed cluster upon drug treatment (23), strongly supporting the application of GEMMs to understand mechanisms of tumorigenesis and concomitantly assessing treatment response at the single-cell level.

Here, we profiled tumors arising in GEMMs carrying the EGFR T790M/L858R mutations and healthy lungs from wild-type EGFR sibling mice, to identify both healthy and malignant cells populating the pulmonary normal and diseased milieus. Such an approach represents a standard of how genetic mutations distress the pulmonary environment at the single-cell level resolution. We identified four main subpopulations, that is, epithelial cells, endothelial cells, fibroblasts, and immune cells, whose overall distribution significantly changes in ADC tissues. Among them, we found both healthy-enriched (C5^epi^, C7^epi^, and C9^epi^), and tumor-specific (C0^epi^ and C4^epi^) AT2 clusters. C0^epi^ and C4^epi^ represent the CSC compartment (STEMMED), as suggested by STREAM analysis that placed them as the first two states (S0 and S1, respectively) in the AT2/AT1 hierarchy. STEMMED showed activation of the GO category “Stem Cell Proliferation” and the pathways typically activated in tumors such as PI3K/AKT, mTOR, JAK/STAT. Interestingly, EGFR signaling was active in STEMMED and downregulated upon treatment with Unesbulin, but only in C4^epi^ and not in C0^epi^. C0^epi^ maintained EGFR signaling activation, as expected with the continuous doxycycline exposure in the mouse model. Our evidence strongly suggests that C0^epi^ is the tumor-initiating cluster (START) that contributes to EGFR T790M/L858R pulmonary tumors. This is the first *in vivo* transcriptional evidence that, similarly to KRAS-mutant AT2 cells (56), AT2 cells carrying EGFR mutations act as lung cancer cells of origin (57).

If transformed epithelial cells in NSCLC are poorly understood at the single-cell level, even less is known about the cancer-associated endothelial cells and fibroblasts (CAF). Despite the well-established general concept that the interactions occurring between tumor cells and endothelial cells are crucial for tumor angiogenesis (58), and that CAFs play protumorigenic roles in altering matrix production and tumor mechanics (59), this study defines the first single-cell mapping of such populations in mutant EGFR-driven ADCs. We identified distinct clusters of endothelial cells and fibroblasts in healthy and tumor tissues. Among them, C6^endo^ and C25^fibro^ were the most prevalent in the TME, representing 86.59% and 93.60% of the endothelial and fibroblast cells, respectively.

It is well known that cancer cells and the host immune response interact in complex ways, and that impaired resolution of inflammation can lead to tumor progression (60). In our dataset, the immune component was dominated by macrophages. Thus, we wondered whether they also have a role in the TME that supports the growth of EGFR-mutant ADCs. Macrophages are abundant in tumor tissues and represent a major component of the tumor-associated inflammation (61). Here, we classified the macrophage clusters into two subtypes: tissue-resident macrophages (AM) and IM that originate from the circulating monocytes.

After characterizing epithelial, endothelial, fibroblast, and macrophage clusters, we analyzed their dynamic cross-talk and how it changes upon drug treatment. Particularly, we focused on the tumor-specific interactions occurring between START, the epithelial target of transformation events, and the other main cell subpopulations (endothelial cells, fibroblasts, and macrophages). Thus, our study provides a comprehensive single-cell atlas of the cell-intrinsic mechanisms and the cell-cell communications that shape the EGFR^TL^ ADC microenvironment. As a result, we show an altered TME with increased proliferation and invasiveness in the malignant epithelial cell compartment, acquisition of TAM properties in the macrophages, coupled to enhanced angiogenesis and ECM dysregulation.

Ligands expressed on the signal-sending cells have an impact on the signal-receiving cell. However, ligands can also exert different effects depending on the receptor they interact with, which is expressed on the receiving cell. This adds an additional level of complexity to cell signaling. Given such a scenario, we performed a systematic analysis of the complex interactions between the par excellence CSC cluster (START) and their melded TME components, to understand the functional mechanisms of lung tumor pathology. Through our analyses, we uncovered specific diseased interactions.

The tumor-specific axes between START and tumor-infiltrating macrophages resulted in signals that support tumor growth and metastasis in the epithelial cells, and acquisition of TAM properties and protumoral phenotypes in the macrophages, creating a cancer-promoting loop. Among the molecules involved in this cross-talk, we identified the GAS6/AXL/MERTK axis. One of the hallmarks of cancer is the immunosuppression of the TME. Indeed, the GAS6/AXL signaling pathway has been implicated in the promotion of immunosuppressive TMEs and immune evasion (62). Furthermore, AXL and MERTK receptors are expressed by TAMs and lead to secretion of immunosuppressive cytokines. Among them, CXCL2 was found to be secreted by M2-TAM in gastrointestinal stromal tumor TMEs and led to tumor metastasis (63). Interestingly, in line with this result, we found CXCL2/CXCR2 axis between both AM, IM, and START, which was completely abolished upon Unesbulin exposure.

C4^macro^ abundance increased during drug treatment, suggesting it may be the IM subtype most involved in mediating the antitumor effect of Unesbulin. IMs also shut down the progranulin (GRN)/sortilin (SORT1) axis, during drug treatment. GRN is a secreted cytokine found to affect CSCs in breast cancer, besides being involved in therapy resistance in a range of cancer types. GRN is a potent CSC activator, and its exposure causes dedifferentiation, as well as increased proliferation of the CSC pool, a process that was shown to be dependent on its receptor SORT1 (64). This evidence is in line with our data showing expression of Sort1 by START.

Both AMs and IMs also manage to down tone (and in some instances even abolish) signaling involving CD44 and several of its ligands. CD44 functions as a receptor for multiple ECM components, as well as a cofactor for growth factors and cytokines, thus, acting as a signaling platform that integrates cellular microenvironmental cues with growth factor and cytokine signals and transduces signals to regulate cell-matrix adhesion, cell migration, proliferation, differentiation, and survival. Liu and colleagues suggested that CD44 is a key regulator of tumor macrophage infiltration, and it may be involved in M2 protumor polarization in bladder cancer (65). To be noted, accumulating evidence indicates that CD44, especially CD44v isoforms, are CSC markers and critical players in regulating the properties of CSCs, including self-renewal, tumor initiation, metastasis, and chemoradioresistance (66). Our data show indeed activation of CD44 on START in the cross-talk involving endothelial and fibroblasts.

The multiple cross-talk involving CD44 identified in the EGFR-mutant TME are linked to Collagen signaling pathways, that are frequently activated by stromal, endothelial cells, and fibroblasts. In pancreatic ductal ADC, this signaling promotes tumor proliferation, migration, and invasion. Col1A1+ tumor-associated endothelial cells promote angiogenesis and neovascularization in PDAC (67).

Malignant AT2 cells secrete ligands that hijack the fibroblasts that subsequently produce abnormal scaffolding (ECM) that allows the tumor to spread. However, Unesbulin can jam such altered communication contributing to blocking tumor growth. A relevant example is represented by GAS6/AXL signaling, that we observed as shut down in macrophages after drug treatment. GAS6/AXL is active also between START and tumor-enriched C25^fibro^ (the major CAF subtype in EGFR-mutant ADCs) until treatment with Unesbulin stops it completely. While AXL expression on cancer cells is readily recognized, it is less well known that AXL is expressed by a variety of host cells found in the TME, including several immune cell types, fibroblasts, osteoclasts, and endothelial cells (62). Our data instead, points to AXL being a potentially relevant player in EGFR-mutant TMEs. AXL may contribute to an immunosuppressive TME and immune evasion of START, and Unesbulin reverses this effect by activating the immune system and enhancing the antitumor response. As a result, AXL is a novel attractive hub for EGFR-mutant NSCLC.

Among the different ECM components, FN1 has been reported as a main driver of tumor progression by different mechanisms (68). FN1 is upregulated in the metastatic niche when lung as well as melanoma cell lines are implanted in lung (69). FN1–CD44 interaction has been shown to promote tumor growth, invasion, and metastasis in breast cancer and glioblastoma (68, 70). In our study, the axis FN1/CD44 is completely lost between C25^Fibro^ and START following drug treatment, similarly to its silencing observed between START and IMs. Concomitantly, numerous interactions related to the binding of CD44 to various types of collagen and laminin, are lost. Because its interaction with ECM ligands promotes invasiveness (71), we propose that the ability of Unesbulin to eliminate them is an antitumorigenic effect. Similarly, most of the interactions that are decreased by Unesbulin are related to syndecans, which are transmembrane heparan sulfate proteoglycans that bind to various ECM proteins and growth factor receptors. Modulating the activity of ECM molecules (collagens, fibronectin, laminins), which are highly expressed in solid tumors, can decrease tumor's growth, invasion, and even resistance development. Therefore, understanding the ECM features is essential to develop strategies to counteract malignancy, given ECM may encapsulate clusters of tumor cells, act as a barrier, and favor resistance (72).

Tumor cells are known to actively enhance vasculature by inducing sprouting of existing vessels, which, in turn, supply tumor cells with survival factors, by secreting VEGF (73). Our data revealed that secretion of multiple ligands by macrophages, endothelial cells, and fibroblasts converge on the same receptors, such as SDC1 and SDC4 on tumor cells. SDC1 participates in establishing a permissive lung microenvironment for breast cancer metastasis (74), while SDC4 engages with EGFR to sustain cell cycle progression in head and neck carcinoma (75). Thus, our data strongly support that the signaling coming from the TME and converging on SDC1 and SDC4 on START tumor cells sustain tumor progression.

CSCs exist in a dynamic equilibrium with their intermingled TME that actively affects their behavior (76) by releasing soluble mediators. Understanding how this niche nurtures CSCs will facilitate novel therapeutic interventions. We found that Unesbulin, an interventional orally bioavailable drug that targets a broad range of cancer types with mild to moderate side effects (77), currently in clinical trial (NCT02404480), effectively downregulated or eliminated the majority of tumor-specific signaling axes we uncovered in the EGFR^TL^ milieu. Unesbulin acts as a potential antitumor agent by interfering with the TME and the CSC niche.

Unesbulin was originally identified by its ability to inhibit proliferation of CSCs expressing the BMI-1 oncoprotein (24). Recently, the downregulation of BMI-1 protein levels and function has been demonstrated to occur in parallel with the potent induction of G_2_–M mitotic arrest and apoptosis, through the inhibition of tubulin polymerization by Unesbulin (47). This evidence is in line with our findings, as we observed that the EGFR-mutant NSCLC cell line H1975 accumulated in G_2_–M, slowed down its in culture proliferation ability, and showed tumor growth inhibition in xenograft models, upon treatment with Unesbulin. We confirmed the BMI-1 hyperphosphorylation and its subsequent degradation, as well as concomitant tubulin degradation, by Western blot analysis. On the basis of these observations, we tested whether anti-BMI-1 treatments could represent a good therapeutic *in vivo* option for BMI-1^+^ EGFR-mutant transgenic models of NSCLC. As expected, after 1 month of treatment, the tumors treated with Unesbulin stopped growing, as compared with the Vehicle-treated tumors, according to the MRI images quantification. Therefore, we adopted the EGFR^TL^ mice as a comprehensive model to capture the cellular populations constituting the tumor environment, their interactions, and their transcriptional changes during drug response. Our scRNA-seq data provide evidence that the population being almost exclusively affected upon drug treatment is the malignant AT2-like compartment. We hypothesized that these cells undergo G_2_–M arrest, tubulin depolymerization, and subsequent apoptosis. This cellular response was accompanied by a concomitant overall decrease of their signaling toward the other cellular compartments populating the TME, and an overall reduction of cross-talk events from the TME to the tumor cells. In accordance with the MRI demonstrating tumor arrest, we hypothesize that attenuating the signaling between the tumor cells and the other TME-embedded cells interfered with the proliferation/aggressive signals that mediate the cross-talk between tumor cells and the TME. Of note, the “Bmi-1 targets” category, which is highly activated both in STEMMED and START in sham-treated tumors, was inhibited upon Unesbulin treatment, as shown by GSEA. Importantly, we also provided evidence that the healthy epithelial cells are not affected by Unesbulin treatment, demonstrating we are specifically targeting tumor cells.

In this article, we have provided important clues to exploit the TME at the single-cell level, to identify novel therapeutic avenues for EGFR-mutant NSCLC. Our findings reveal the complex cell-cell communication and signaling dynamics that occur between malignant AT2 cells and other cell types embedded in the tumor setting. These insights may help to understand the molecular mechanisms and cellular interactions that underlie lung cancer development and progression. Moreover, our data support the preclinical use of GEMMs to identify cellular and molecular vulnerabilities, and encourage development of future Unesbulin-based therapies that may be tested in the notable percentage of patients with NSCLC carrying EGFR mutations.

## Supplementary Material

Supplementary Figure S1 Supplementary Figure S1. BMI-1 inhibition affects cell cycle progression and tumor growth

Supplementary Figure S2 Supplementary Figure S2. Single-cell RNA sequencing cluster distribution in healthy lungs

Supplementary Figure S3 Supplementary Figure S3. Deconvolution of healthy and diseased epithelial clusters highlights the malignant nature of C0epi and C4epi

Supplementary Figure S4 Supplementary Figure S4. Deconvolution of healthy and diseased macrophage clusters demonstrates presence of interstitial and alveolar subpopulations

Supplementary Table 1 Supplementary Table 1. Size of each identified transcriptional cluster as percentage of all profiled cells.

Supplementary Table 2 Supplementary Table 2. Size of each identified transcriptional cluster as percentage of all profiled cells.

Supplementary Table 3 Supplementary Table 3. Size of each identified transcriptional cluster as percentage of all profiled cells.

Supplementary Table 4 Supplementary Table 4. Size of each identified cell type as a percentage of all profiled cells.

Supplementary Table 5 Supplementary Table 5. Size of each identified cell type as a percentage of all profiled cells. P values are indicated.

Supplementary Table 6 Supplementary Table 6. Size of each identified transcriptional cluster as percentage of all profiled cells.

Supplementary Table 7 Supplementary Table 7. Size of each identified transcriptional cluster as percentage of all profiled cells.

Supplementary Table 8 Supplementary Table 8. Size of each identified cell type as a percentage of all profiled cells.

Supplementary Table 9 Supplementary Table 9. Size of each identified cell type as a percentage of all profiled cells.

Supplementary Table 10 Supplementary Table 10. Size of each identified transcriptional cluster as percentage of all profiled cells.

Supplementary Table 11 Supplementary Table 11. Size of each identified transcriptional cluster as percentage of all profiled cells.

Supplementary Table 12 Supplementary Table 12. Size of each identified transcriptional cluster as percentage of all profiled endothelial cells.

Supplementary Table 13 Supplementary Table 13. Size of each identified transcriptional cluster as a percentage of all profiled endothelial cells.

Supplementary Table 14 Supplementary Table 14. Size of each identified transcriptional cluster as percentage of all profiled fibroblasts.

Supplementary Table 15 Supplementary Table 15. Size of each identified transcriptional cluster as a percentage of all profiled fibroblasts.
