# Correction: Tumor Microenvironment Landscapes Supporting EGFR-mutant NSCLC Are Modulated at the Single-cell Interaction Level by Unesbulin Treatment

**DOI:** 10.1158/2767-9764.CRC-25-0200

**Published:** 2025-04-18

**Authors:** Giorgia Maroni, Indira Krishnan, Roberta Alfieri, Valerie A. Maymi, Nicole Pandell, Eva Csizmadia, Junyan Zhang, Marla Weetall, Art Branstrom, Giulia Braccini, Eva Cabrera San Millán, Barbara Storti, Ranieri Bizzarri, Olivier Kocher, Daniela S. Bassères, Robert S. Welner, Maria Cristina Magli, Ivan Merelli, John G. Clohessy, Azhar Ali, Daniel G. Tenen, Elena Levantini

In the original version of this article (1), the name of the 15th author, Daniela S. Bassères, was incorrect. The name has been corrected in the latest online HTML and PDF versions of the article. The publisher regrets this error.
