# Supplementary material for: Tumor Microenvironment Landscapes Supporting EGFR-mutant NSCLC Are Modulated at the Single-cell Interaction Level by Unesbulin Treatment
Source: Cancer Res Commun. 2024 Mar 26;4(3):919–37. doi: 10.1158/2767-9764.CRC-23-0161 (PMC10964845; doi:10.1158/2767-9764.CRC-23-0161)
Supplement: Supplementary Figure S1 — BMI-1 inhibition affects cell cycle progression and tumor growth [file crc-23-0161-s01.docx]

Supplementary Figure S1


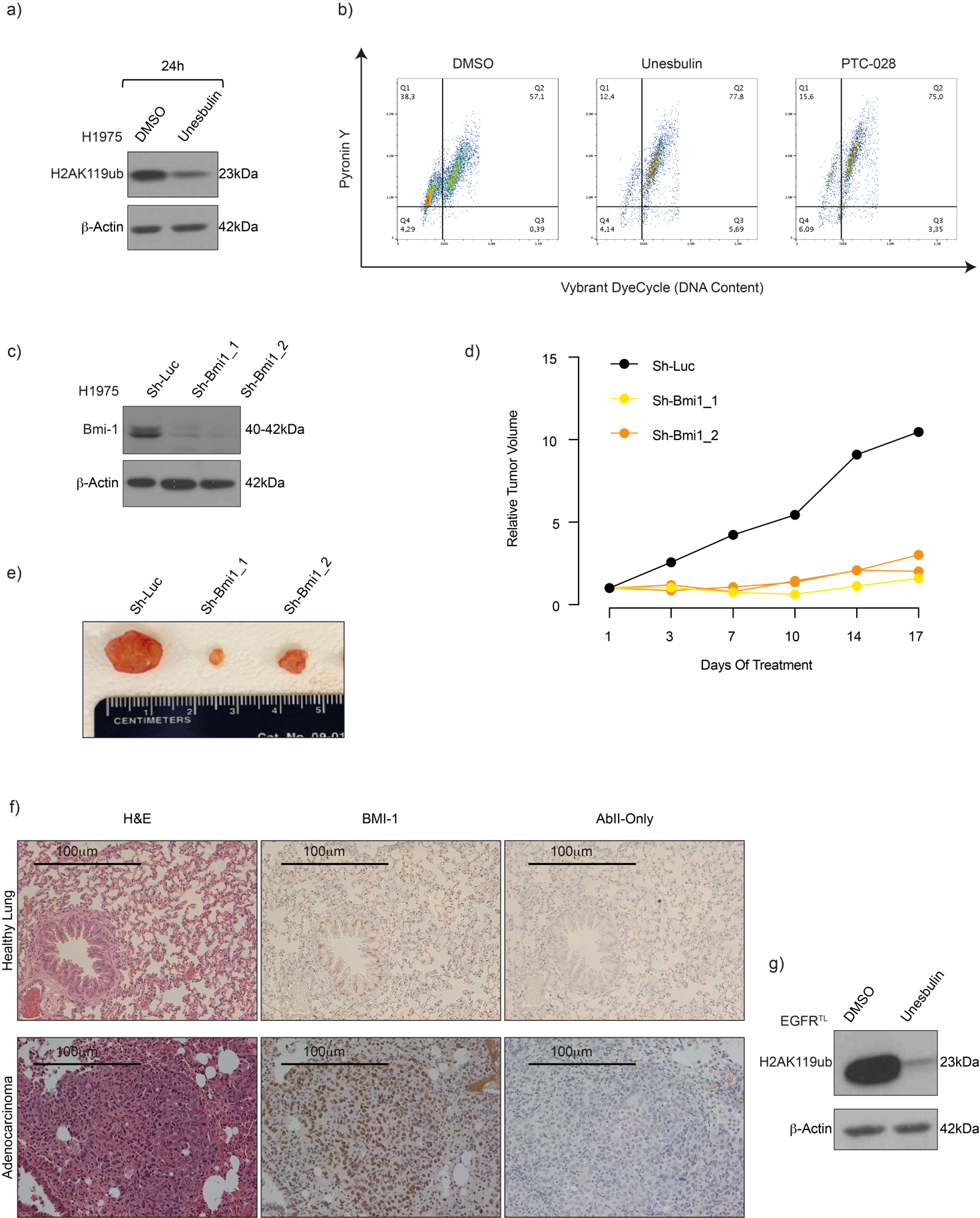


**Suppl. Fig. S1. BMI-1 inhibition affects cell cycle progression and tumor growth**

**a** Western blot analysis of the human H1975 cell line treated for 24 hours with DMSO as control and Unesbulin. Protein lysates were immunoblotted with an anti-H2AK119ub antibody. Loading was assessed with an anti-β-actin antibody. Expected size is shown in kDa. **b** Cell cycle analysis of the H1975 cell lines after treatment for 24 hours with DMSO (left panel), Unesbulin (middle panel) or PTC-028 (right panel). The gating shows distribution of cells in G_0_ (in Q4), G_1_ (in Q1), and G_2_-M (in Q2) phases. Representative percentages of cells in each cell cycle phase are indicated. **c** Western blot analyses of the human H1975 cell lines transduced with control shRNA (Sh-Luc) or two shRNAs against BMI-1 (Sh-Bmi1_1 and Sh-Bmi1_2). Protein lysates were immunoblotted with an anti-BMI-1 antibody. Loading was assessed with an anti-β-actin antibody. Expected size is shown in kDa. **d** NSG mice were subcutaneously injected with Sh-Luc-transduced H1975 cells (n=1), or Sh-Bmi1_1- (n=1) or Sh-Bmi1_2-transduced H1975 cells (n=2). The figure indicates tumor volume versus time of treatment (in days). **e** Picture of tumors growing subcutaneously in NSG mice after injection of cells infected with control Sh-Luc, or Sh-Bmi1_1 and Sh-Bmi1_2. **f** Representative histological lung sections of healthy lungs (upper row) and tumors from EGFR^TL^ mice (bottom row), stained with hematoxylin and eosin (left panels), anti-BMI-1 antibody (middle panel), and secondary-only antibody, as control (right panels). Scale bar, 100 μm. **g** Western blot analyses of *in vivo* tumors from EGFR^TL^ mice, treated with DMSO or Unesbulin, at treatment termination. Protein lysates were immunoblotted with an anti-H2AK119ub antibody. Loading was assessed with an anti-β-actin antibody. Expected size is shown in kDa.
