# Supplementary material for: Tumor Microenvironment Landscapes Supporting EGFR-mutant NSCLC Are Modulated at the Single-cell Interaction Level by Unesbulin Treatment
Source: Cancer Res Commun. 2024 Mar 26;4(3):919–37. doi: 10.1158/2767-9764.CRC-23-0161 (PMC10964845; doi:10.1158/2767-9764.CRC-23-0161)
Supplement: Supplementary Figure S2 — Single-cell RNA sequencing cluster distribution in healthy lungs [file crc-23-0161-s02.docx]

Supplementary Figure S2


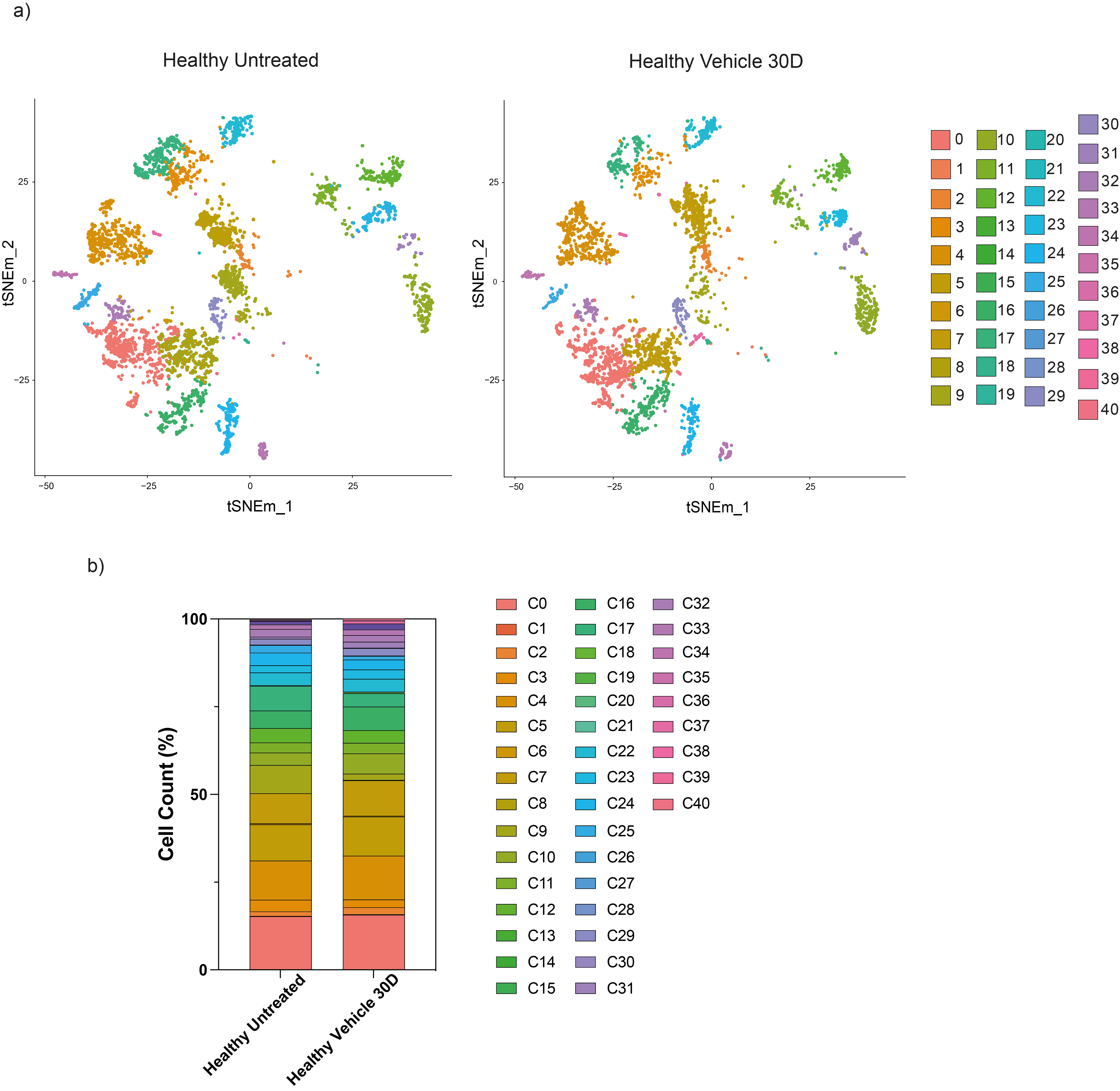


**Suppl. Fig. S2. Single-cell RNA sequencing cluster distribution in healthy lungs**

**a** t-SNE plot of the 41 clusters identified in untreated (n=2, left panel), and Vehicle-treated (n=3, right panel) healthy lungs. Each point represents one cell. Each color represents a defined transcriptional cluster as shown in the corresponding legends. **b** Histograms representing the percentage contributions of C0-C40 clusters per sample (Untreated and Vehicle-treated healthy lungs).
