# Supplementary material for: Tumor Microenvironment Landscapes Supporting EGFR-mutant NSCLC Are Modulated at the Single-cell Interaction Level by Unesbulin Treatment
Source: Cancer Res Commun. 2024 Mar 26;4(3):919–37. doi: 10.1158/2767-9764.CRC-23-0161 (PMC10964845; doi:10.1158/2767-9764.CRC-23-0161)
Supplement: Supplementary Figure S3 — Deconvolution of healthy and diseased epithelial clusters highlights the malignant nature of C0epi and C4epi [file crc-23-0161-s03.docx]

Supplementary Figure S3


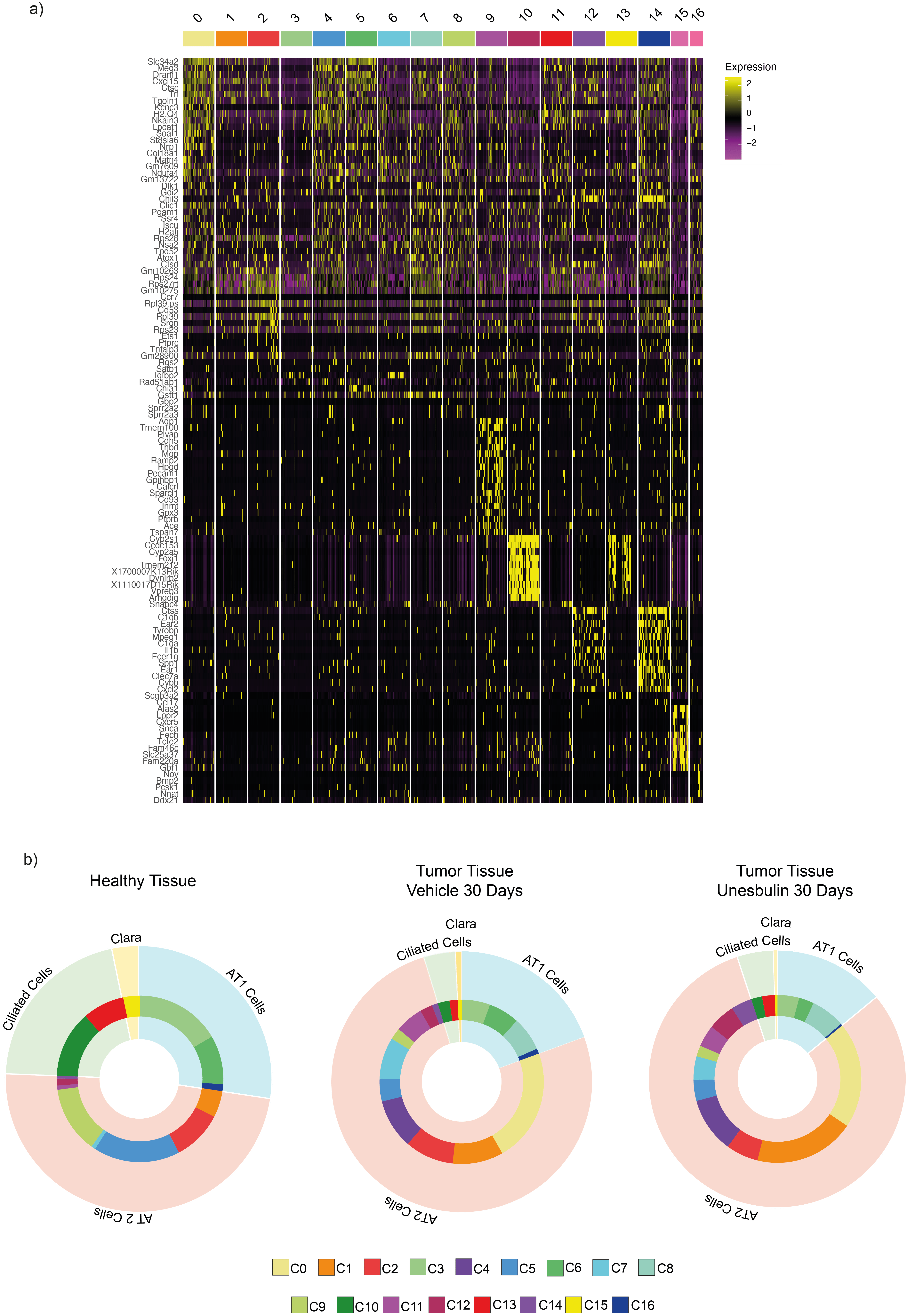


**Suppl. Fig. S3. Deconvolution of healthy and diseased epithelial clusters highlights the malignant nature of C0^epi^ and C4^epi^**

**a** Heatmap showing marker genes of each defined epithelial cluster. **b** Cluster distribution superimposed to clusters annotation in epithelial cells of healthy lungs (left panel), and Vehicle- (middle panel) or Unesbulin-treated tumors (right panel).
