# Supplementary material for: Tumor Microenvironment Landscapes Supporting EGFR-mutant NSCLC Are Modulated at the Single-cell Interaction Level by Unesbulin Treatment
Source: Cancer Res Commun. 2024 Mar 26;4(3):919–37. doi: 10.1158/2767-9764.CRC-23-0161 (PMC10964845; doi:10.1158/2767-9764.CRC-23-0161)
Supplement: Supplementary Figure S4 — Deconvolution of healthy and diseased macrophage clusters demonstrates presence of interstitial and alveolar subpopulations [file crc-23-0161-s04.docx]

Supplementary Figure S4


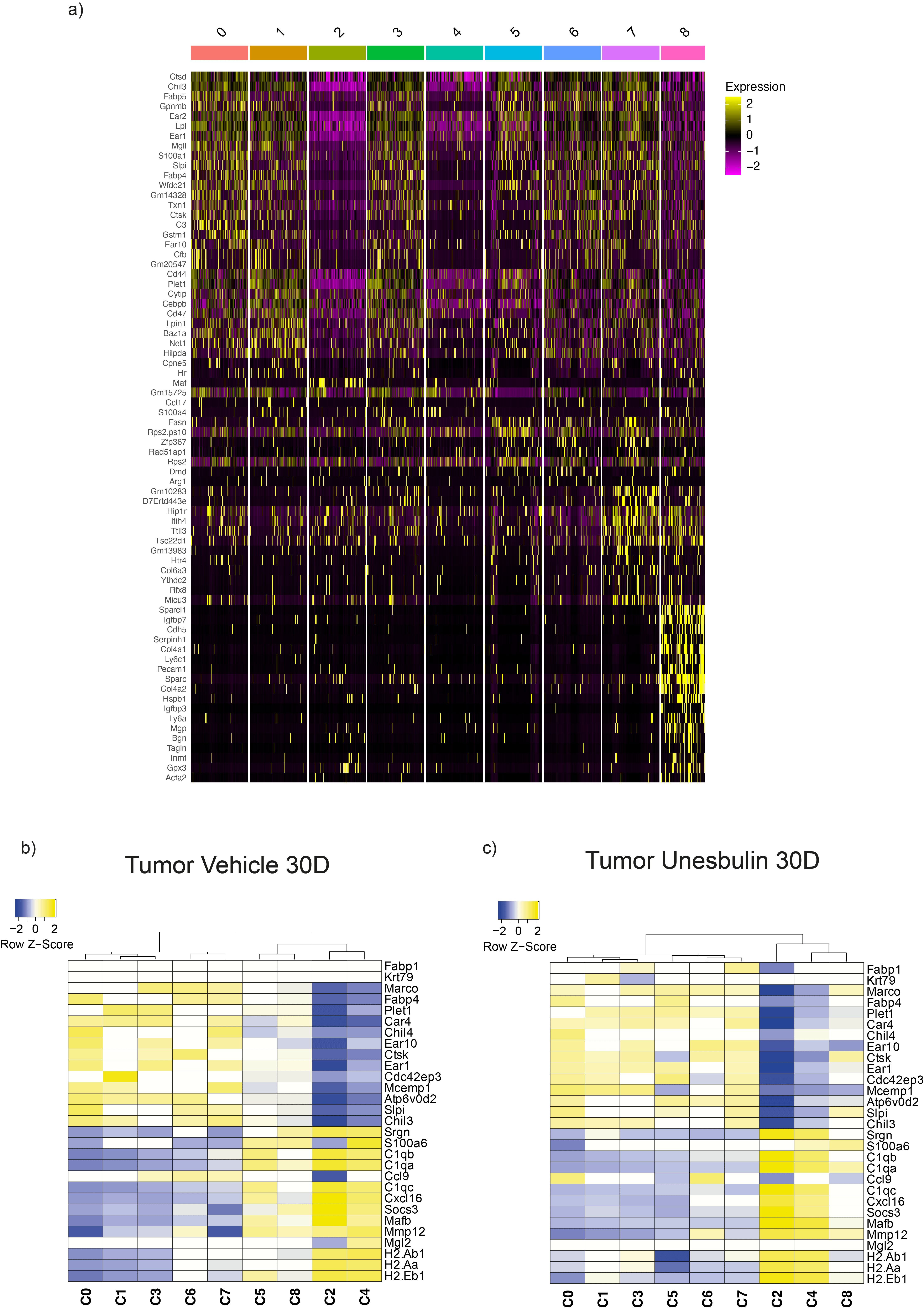


**Suppl. Fig. S4. Deconvolution of healthy and diseased macrophage clusters demonstrates presence of interstitial and alveolar subpopulations**

**a** Heatmap showing the marker genes in C0-C8 clusters. Heatmap showing selected alveolar and interstitial macrophage expressed genes in Vehicle-treated (**b**) and d Unesbulin-treated EGFR^TL^ macrophage clusters (**c**).
