# Supplementary material for: Tumor Microenvironment Landscapes Supporting EGFR-mutant NSCLC Are Modulated at the Single-cell Interaction Level by Unesbulin Treatment
Source: Cancer Res Commun. 2024 Mar 26;4(3):919–37. doi: 10.1158/2767-9764.CRC-23-0161 (PMC10964845; doi:10.1158/2767-9764.CRC-23-0161)
Supplement: Supplementary Table 9 — Size of each identified cell type as a percentage of all profiled cells. [file crc-23-0161-s13.docx]

**Supplementary Table 9.**

Size of each identified cell type as a percentage of all profiled cells. P values are indicated.
